# The effect of identity-related interventions on physical activity- and smoking-related identities and behavior: a mixed-methods systematic review

**DOI:** 10.1186/s13643-026-03103-2

**Published:** 2026-02-24

**Authors:** Milon H. M. van Vliet, Kristell M. Penfornis, Winifred A. Gebhardt, Emma F. von Haeseler, Niels H. Chavannes, Anke Versluis, Eline Meijer

**Affiliations:** 1 Department of Public Health and Primary Care, Leiden University Medical Centre, Leiden, The Netherlands; 2 National eHealth Living Lab, Leiden University Medical Centre, Leiden, The Netherlands; 3 Unit Health-, Medical and Neuropsychology, Institute of Psychology, Leiden University, Leiden, The Netherlands

**Keywords:** Smoking, Physical activity, Identity, Identity-related interventions, Systematic review, Mixed methods

## Abstract

**Background:**

Identity, representations individuals hold about themselves, drives health behavior change, influencing both health-promoting and health-compromising behaviors. This mixed-methods systematic review synthesizes interventions directly targeting physical activity (PA)- and/or smoking-related identities to promote PA and smoking cessation in individuals aged 12 years and over. It also examines the effects of these interventions on identity and behavior, as well as mediators or moderators of effectiveness.

**Methods:**

A comprehensive search across nine databases identified 5801 records, with 19 reports (20 studies) being included. Two independent reviewers extracted data and assessed study quality using the Mixed Methods Appraisal Tool.

**Results:**

Four types of identity-related interventions were identified: possible-self interventions, multi-component interventions incorporating identity components, possible-self avatar games, and identity-challenge interventions. Intervention effectiveness was mixed: nearly half of the studies reported significant positive effects on PA- and smoking-related identities and behaviors, while others found no significant differences. No significant negative effects were reported. Although results seem similar for both behaviors, more studies focused on PA, complicating direct comparisons. One study suggested that integrating PA promotion and smoking cessation into a single intervention could be beneficial, but further research is needed. Identified mediators and moderators include self-efficacy, planning, and consideration of future consequences.

**Conclusions:**

Overall, identity-related interventions show promise for PA promotion and smoking cessation, but optimal design and operationalization remain uncertain. Tailoring these interventions to individual characteristics may enhance effectiveness and contribute to public health.

**Systematic review registration:**

Open Science Framework https://osf.io/avtx4/

**Supplementary Information:**

The online version contains supplementary material available at 10.1186/s13643-026-03103-2.

## Background

Identity is important in regulating behavior [1–6]. Most identity theories define it as a mental representation that individuals hold about themselves [2–4]. A distinction can be made between individual-level and interpersonal-level identities [5, 7]. Individual-level identities, such as self-identity, are conceptualized as based on one’s self-perception as an individual (e.g., “parenting fits with who I am”) [2, 3, 5]. Interpersonal-level identities, such as social identity, are conceptualized as based on identification with a social category or group to which one perceives oneself as belonging (e.g., feeling connected to other parents) [3, 5, 8]. Many theories of identity, such as the Identity Theory [2], state that people tend to act in line with their most salient identities. Consequently, identity, and even projected future identity, are theorized to be important determinants of behavior change, as supported by empirical reviews [1, 5, 6]. To illustrate, Possible Selves Theory (PST) posits that an individual can have multiple possible future identities (i.e., possible selves) representing one’s hopes, fears, goals, and threats [9]. People are assumed to have both desired future self-representations (e.g., becoming a manager) and undesired future self-representations (e.g., becoming unemployed). According to PST, people tend to act in ways that will bring them closer to becoming their desired future selves and help them avoid their undesired future selves [9, 10]. In this way, identity can guide behavior, and influence behavior change.

Influencing identities is a promising strategy for changing unhealthy behaviors, such as low physical activity (PA) and tobacco smoking. Addressing these two health behaviors is a major public health priority due to their substantial impact on non-communicable diseases, mortality, healthcare costs, and health inequity [11–15]. Smoking, as the leading risk factor for mortality, accounts for 20.2% of male deaths and 5.8% of female deaths globally [13], while physical inactivity accounts for 7.2% of deaths [11]. Health-related identities can drive both health-promoting and health-compromising behaviors. PA promotion and smoking cessation provide compelling examples: previous reviews and meta-analyses show that a stronger PA-related identity is associated with higher intentions to engage in PA [5, 6] and increased PA levels, while a stronger physical inactivity-related identity is linked to lower PA levels [5]. Similarly, smoking-related identities are associated with smoking behavior. Specifically, holding a stronger “smoker” identity is linked to continued smoking, whereas a stronger “non-smoker” or “ex-smoker” identity is associated with quitting and maintaining abstinence [1, 5, 16]. These relationships between PA- and smoking-related identities and behaviors, respectively, were found to endure over time [5, 6]. This suggests that identity may not only be important for initiating health behavior change, but also for maintaining it. Therefore, identity-related interventions have gained increased interest over the last two decades for PA promotion and smoking cessation.

Health-related identity interventions directly target identity to promote behavior change by strengthening individuals’ healthy identities and/or weakening unhealthy identities. For example, in possible-self interventions, individuals are prompted to consider their healthy possible-self (e.g., exerciser) [17, 18]. Previous studies have investigated possible-self interventions in the context of PA [17, 19] and smoking [20, 21]. showing mixed results. Identity interventions can also be included in multi-component behavior change interventions (e.g., [22]). To illustrate, a systematic review focusing on behavior change techniques used in multi-component smoking cessation interventions found that prompting identity associated with changed behavior is a behavior change technique predictive of higher smoking cessation rates [23]. All in all, previous research highlights the potential of identity-related interventions for PA promotion and smoking cessation. Providing an up-to-date and comprehensive summary of the current knowledge on interventions that directly target identity can provide insight into their effectiveness and clarify the conditions under which they are most effective.

Investigating interventions targeting PA and smoking in tandem is valuable, as such unhealthy behaviors often co-occur [24, 25] and are inversely associated, both behaviorally [26–30] and in terms of related identities [30]. That is, engaging in PA may mitigate smoking cravings [26, 27], increase motivation to quit smoking [28], and reduce psychological distress [27], making successful smoking cessation more likely [26]. Conversely, smoking cessation can improve fitness, which could facilitate PA engagement [29] Furthermore, not only PA behavior but also PA-related identity is suggested to be negatively related to smoking in adolescents [30]. Additionally, a parallel scoping review [5] (following the same protocol [31]) shows many similarities between identity processes in the context of PA and smoking, indicating commonalities in identity mechanisms underlying health-promoting and health-compromising behaviors. By targeting PA- and smoking-related identities together, interventions may leverage these shared identity mechanisms to amplify behavior change across domains. Therefore, exploring whether there are identity-related interventions targeting both behaviors, their effectiveness, and the conditions under which they are most effective is highly relevant.

To our knowledge, only a few reviews have investigated the influence of interventions on identity in health contexts. These include one rapid evidence assessment on the effectiveness of interventions targeting personal or social identity [1], one meta-analysis on best possible-self interventions for wellbeing [32], one systematic review examining the influence of possible selves on health behaviors in adolescents [33], and one recent meta-analysis investigating the effect of PA interventions on PA identity [34]. However, no systematic review has yet provided a comprehensive synthesis of different types of identity-related interventions targeting *both* PA promotion and smoking cessation in individuals aged 12 years and older. This highlights a critical gap in understanding the broader applicability and potential synergies of these interventions. Additionally, the current mixed-methods systematic review specifically focuses on interventions that directly target PA- or smoking-related identities using identity behavior change techniques. Unlike, for example, the review by Rhodes et al. [34], which includes both identity-related and general PA interventions, our review exclusively examines interventions that directly target identity. By isolating interventions directly aiming to influence identity, this review provides insight into their effectiveness and conditions for success.

Variables such as age [5, 16, 35], gender [5, 36], and PA- or smoking history [5, 37, 38] have been shown to influence PA- and smoking-related identity, and could also influence the effectiveness of identity-related interventions and make interventions more suitable for certain populations. Hence, summarizing findings on mediating and moderating factors of the effects of identity-related interventions is also important to inform the future development and operationalization of these interventions.

The first aim of this mixed-methods systematic review was to describe identity-related interventions using identity behavior change techniques to promote PA (i.e., a health-promoting behavior) and smoking cessation (i.e., a health-compromising behavior) in individuals aged 12 years and over. We focused on populations aged 12 years and over because identity typically starts forming around this age and continues to develop throughout adolescence into adulthood [39]. Second, we aimed to summarize the effects of these interventions on PA- and smoking-related identities, and/or the promotion of PA and smoking cessation behaviors. Third, we examined mediators or moderators of the effectiveness of identity-related interventions in the context of PA and smoking. Through this work, we aim to provide recommendations for future researchers on developing these interventions and to identify current knowledge gaps that require further research.

## Methods

This mixed-methods systematic review was conducted and reported in accordance with the Preferred Reporting Items for Systematic Reviews and Meta-Analyses (PRISMA) guidelines (see Additional file 1) [40]. The review process followed our protocol, which was prospectively registered on the Open Science Framework (OSF; https://osf.io/avtx4/) and detailed in a published report [31]. Initially, the review was conceived as a scoping review addressing two broad research questions: (1) what is known about identity (processes) in the context of PA and smoking, taking into account demographic, PA- and smoking-specific characteristics?; and (2) What identity-related interventions are used to influence PA and smoking, taking into account demographic, PA- and smoking-specific characteristics? [31]. However, after the title and abstract screening phase, we decided to split the review into two parts: a scoping review to investigate the first research question [5] and the current systematic review to investigate the second research question. This decision was made because the literature search yielded more relevant literature than anticipated, allowing for a more in-depth examination of each question. As the methods are described in detail in the published review protocol [31], this section will provide only a brief overview, also highlighting any deviations from the protocol.

### Eligibility criteria

Studies were included if they were peer-reviewed, available in full text, and investigated the effect of an identity-related intervention on PA- and/or smoking-related identity, behavior, and/or precursors of behavior in individuals aged 12 years and over (see Additional file 2 for detailed eligibility criteria). All study designs were considered relevant, provided they included an identity-related intervention. Identity-related interventions were defined as interventions employing tasks, manipulations, or exercises directly aimed at influencing PA- and/or smoking-related identity (i.e., using “identity” behavior change techniques as outlined in the behavior change taxonomy v1 of Michie et al. [41]). This definition also includes avatar studies which use avatars as visualized possible selves by comparing a self-avatar with a general other-avatar, and identity-challenge interventions, which assess the reactions of participants with different identity levels to hypothetical identity challenges. Furthermore, multi-component interventions with an identity component (e.g., an intervention combining a possible-self exercise with other behavior change techniques, such as goal-setting) were included. However, multi-component studies that only assessed the intervention’s effect on PA- and/or smoking behavior, without examining changes in related identities, were excluded to avoid confusing the effects of the identity component with those of the other behavior change techniques. There were two minor deviations from the original inclusion criteria [31]. First, the language inclusion criterion was expanded to include studies written in German or any language with a comprehensible and coherent translation available via a translating machine, in addition to English, Dutch, and French. Second, the inclusion criterion regarding participants’ baseline activity levels was broadened to include individuals who engage in any level of PA, rather than only those who are insufficiently active. This change was made because increasing PA is also relevant for individuals who are already active.

Studies were excluded if they were research protocols, study design reports, non-interventional design studies, or literature reviews without individual study results. Additionally, studies were excluded if they did not report sufficient data or results relevant to our research questions (e.g., no statistical comparisons were reported, or polysubstance was studied and it was not possible to identify which findings pertained specifically to smoking). Finally, studies were excluded if they investigated interventions without an identity component (e.g., an aerobics class intervention).

### Search strategy

A comprehensive three-step search strategy was conducted in collaboration with a research librarian (JWS), as outlined in the review protocol [31]. This strategy aimed to identify relevant studies from inception until May 2023. Search strings were used across nine electronic databases: PubMed, Web of Science Core Collection, PsycINFO, Wiley Cochrane Library, Embase, Emcare, PsycArticles, Psychology and Behavioral Sciences Collection, and Academic Search Premier. Search terms related to identity (e.g., self-identity, self-schema, possible selves), PA (e.g., exercise, sports, physical inactivity), and smoking cessation (e.g., smoking, tobacco, abstinence) were used (see Additional file 3 for the full search strings). In addition to database searches, backward and forward reference searching was conducted. After the title and abstract screening, we manually searched for relevant studies linked to dissertations, research protocols, study design reports, and commentaries that were identified during screening but excluded due to the absence of peer review or study results. During the full-text screening, we also reviewed reference lists from relevant literature reviews in the full-text screening sample and searched for studies by key authors in the research field.

### Selection process

After removing duplicates, the titles and abstracts of studies identified through our search strategy were screened for eligibility using machine-learning software ASReview [42] and the screening assistant web application Rayyan [43]. Studies marked as “included” were subsequently screened for eligibility based on their full texts. A detailed description of the screening software and the two screening phases can be found in the review protocol [31].

There were two deviations from the original review protocol [31]. First, two rounds of title and abstract screening were conducted instead of one. In the first round, the pre-established in- and exclusion criteria were intentionally broad. After screening a sufficient number of titles and abstracts to gain a comprehensive overview of the available literature, these criteria were refined in consultation with the review team, and the remaining titles and abstracts were then screened in a second round. In this round, all records that had passed the first screening, as well as records that had not yet been screened, were screened again using the refined, more stringent in- and exclusion criteria. Second, following the title and abstract screening, the full texts of the remaining studies were obtained and categorized according to their relevance to the parallel scoping review [5] and this systematic review. After categorization, the full-text screening manual, including the in- and exclusion criteria, was refined and applied exclusively to the studies relevant to this systematic review. Reasons for exclusion were documented. These deviations from the original protocol arose mainly because the review initially started as a scoping review, which typically allows for a more flexible and iterative approach [44], but was then split into a parallel scoping review [5] and this systematic review. Screening manuals for both screening phases were iteratively developed and pilot-tested, and can be found in Additional file 2.

For both rounds of title and abstract screening, a random sample of 10% of the titles and abstracts was independently double-screened by two reviewers (KMP and MHMV). Interrater agreement for this phase was moderate (absolute agreement = 86.4% and Cohen’s *Κ* = 0.59) [45, 46], indicating the reliability of the screening process and the machine-learning software ASReview. All full texts were independently double-screened by two reviewers (MHMV and EFH [original search] or AV [search update]). Full-text interrater agreement was moderate to high (absolute agreement = 89.2% and Cohen’s *Κ* = 0.74) [45, 46]. Disagreements were discussed and resolved by consensus, with a third reviewer (EM) involved if necessary.

### Data extraction

Relevant data were systematically extracted from the included studies using a pilot-tested data extraction form in Microsoft Excel. This form was developed in consultation with the review team and pilot-tested a priori by FB and MHMV. The data extraction form captured the following information: general study information (e.g., authors, language), setting and design, type of behavior studied (i.e., smoking, PA, or both), study hypotheses and aims, number and type of relevant measurement instruments, intervention characteristics (e.g., procedure, duration), demographic characteristics of the sample (e.g., sample size, age), PA- or smoking-related characteristics of the sample (e.g., smoking onset, PA level), identity theories underlying the intervention, type of identity targeted, results per relevant outcome (i.e., PA- and smoking-related identities, PA and smoking behavior, precursors of PA and smoking behavior, mediators and moderators), information on missing data and attrition, and study conclusions. Data were extracted and coded by one reviewer (EFH), with a random sample of 25% of the studies independently double-coded by another reviewer (MHMV). Again, disagreements were discussed and resolved by consensus, involving a third reviewer (EM) if necessary. If key data needed to answer our research questions were missing or unclear, authors were contacted via email, with one reminder sent if no response was received.

### Quality assessment

The review protocol [31] originally specified the use of the JBI Critical Appraisal Tools [47] for quality assessment. However, we selected the Mixed Methods Appraisal Tool (MMAT; version 2018) [48] as it combines assessments for different study designs into a single standardized tool. Five types of biases were assessed for qualitative studies, quantitative randomized controlled trials (RCTs), and quantitative non-randomized studies, and 15 types of biases were assessed for mixed-methods studies. Each bias type was rated as “yes” (no indication of bias), “no” (clear indication of bias), or “can’t tell” (unclear due to missing information). We provided a detailed overview of the quality assessment by bias and an overall rating per study. This overall rating ranges from 0% (i.e., all biases rated as “no” or “can’t tell”; lowest rating) to 100% (i.e., all biases are rated as “yes”; highest rating). This rating was also included in the same overview as the individual study findings to help readers interpret findings in the context of study quality. For mixed-methods studies, for which 15 biases were evaluated instead of five, the total number of “yes” ratings was divided by three to calculate the overall rating.

The MMAT was pilot-tested by FB and MHMV, with guidelines for assessing biases detailed and tailored to the type of studies relevant to this review through discussion. One reviewer (EFH) rated the biases for each study, following the MMAT manual and our additional notes. A random sample of 25% of the studies was independently double-coded by another reviewer (MHMV). In cases of uncertainty, EFH also discussed the bias assessments with MHMV. Detailed notes were taken on all bias assessments. Disagreements were resolved through discussion, involving a third reviewer (EM) if necessary.

### Synthesis of results

As only limited qualitative data were available, the qualitative data were narratively integrated at the study level to complement the quantitative results, without formal data transformation or sequential/convergent synthesis. The combined qualitative and quantitative results from the included studies were synthesized narratively and organized according to the three research aims. For the first aim, which focused on describing identity-related interventions for promoting PA and smoking cessation, identity-related interventions with similar characteristics were grouped into distinct categories. For the second aim, which focused on summarizing the effects of these interventions, the synthesis was organized by intervention type, comparing their effects on (1) PA- and/or smoking-related identities and (2) PA and/or smoking behaviors or intentions. For the third aim, which focused on examining mediators and moderators, we synthesized results from the studies that included mediation or moderation analyses to identify factors influencing intervention effects. Differences and similarities between studies were explored and described. Additionally, study characteristics and results were tabulated to create clear and concise summaries to support the narrative text. Categorization and organization of the results were performed by EFH and MHMV, in consultation with the review team.

## Results

**Fig. 1:**
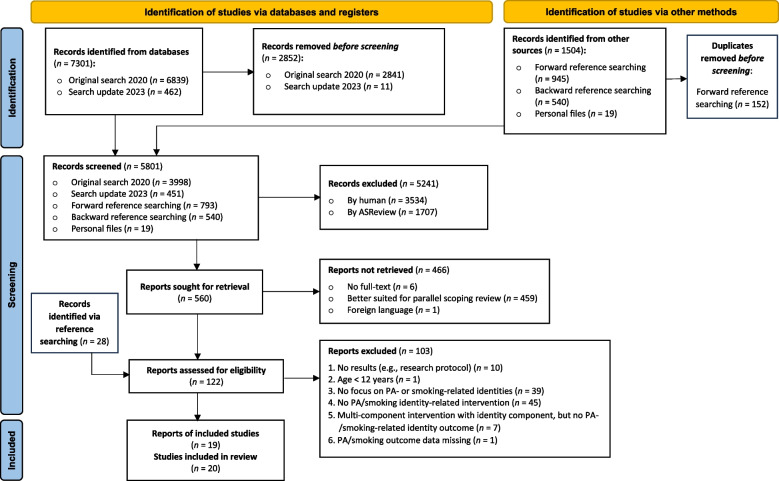
PRISMA diagram of study selection process. *From:* Page MJ, McKenzie JE, Bossuyt PM, Boutron I, Hoffmann TC, Mulrow CD, et al. The PRISMA 2020 statement: an updated guideline for reporting systematic reviews. BMJ 2021;372:n71. https://doi.org/10.1136/bmj.n71

The first part of the study selection process (study identification and title and abstract screening) was conducted jointly for this systematic review and the parallel scoping review [5] (see Fig. 1 for the PRISMA diagram). In total, 8805 reports were identified and, after removing duplicates, 5801 were screened based on title and abstracts. After this, the study selection process was performed separately for both reviews. For this systematic review, 122 full-text reports were screened, including 28 identified through reference searching, resulting in a total of 19 relevant reports included in this review. One of these reports was a secondary analysis [49] of a study described in another report [50], and was therefore counted as one study. Additionally, two reports described two different studies relevant to this review (i.e., studies 2 [51A] and 3 [51B] in Fox and Bailenson [51], and studies 1 [52A] and 2 [52B] in Helweg-Larsen et al. [52]). Therefore, the 19 reports describe a total of 20 different studies. One study initially met all inclusion criteria but was excluded because although PA behavior was mentioned as one of the outcomes, no actual results on PA behavior were reported, and the authors did not respond to our requests for missing data (see exclusion reason “6. PA/smoking outcome data missing” in Fig. 1) [53].

### Study characteristics

The characteristics of the included studies (*n* = 20) are presented in Table 1. The studies were published between 2005 [17] and 2023 [20], all in English. Most studies were quantitative (*n* = 16/20) [17, 19, 20, 54–62], with the rest being mixed-methods studies (*n* = 4/20) [21, 22, 50, 60]. Of the mixed-methods studies, only two provided qualitative results relevant to our research question [21, 50]. Although the selection process allowed for qualitative studies, no relevant qualitative studies emerged from the selection process. Most studies employed a controlled design (*n* = 18/20) [17, 19–22, 54–61, 63], with 72% comparing interventions with an active comparator (e.g., imagery task focused on action planning) (*n* = 13/18) [17, 21, 54, 56, 58–61, 63], 22% with a passive comparator (e.g., only completing study measures) (*n* = 4/18) [19, 20, 22, 58], and one with both [56]. Two studies employed a single-arm pre-post design [50, 64]. Study duration ranged from 1 week [62] to 36 weeks [59] but was unclear for some of the studies (*n* = 6/20) [51AB, 52AB, 61, 62]. More studies investigated identity-related interventions for PA promotion (*n* = 13/20) [17, 19, 22, 54–58, 60, 62, 63] than for smoking cessation (*n* = 6/20) [20, 21, 59, 61]. Only one study targeted both behaviors [50]. Most studies (*n* = 17/20) [17, 19–22, 54, 56–63] used self-report questionnaires to assess the outcomes relevant to this review (e.g., Exercise Identity Scale [67], Godin Leisure-Time Exercise Questionnaire [65]).

**Table 1:** Characteristics of the included studies (*n* = 20)

First author (year) Design, setting, and country	Behavior	Population characteristics	PA/smoking characteristics at baselinefek	Intervention and comparator conditions	Measurements and intervention duration	Results MMAT quality assessment rating^a^
Possible-self (PS) interventions (*n* = 8)						
Chan (2012) [54] Quantitative 2 × 2 factorial design In person New Zealand	PA	*N*_baseline_ = 182 *N*_analysis_ = 136 *M*_age_ = not reported 81.9% female	PA frequency *M* = 101 min of PA a week (insufficiently active^b^)	Interventions Approach imagery (= desired PS) Approach-process imagery (= desired PS + action planning) Active comparators Process imagery (= focused on action planning) Neutral imagery (= focused on performing PA)	T0–baseline T1–post-intervention T2–1-week follow-up T3–2-week follow-up T4–4-week follow-up Type of measurement Self-report Frequency^c^ Single session Duration Intervention: ~ 5 min^c^ Study: 4 weeks	PA precursor + * Intention to engage in PA significantly increased within all conditions over time. PA intention increased more for the approach imagery (= desired PS condition), compared to the process imagery and neutral control condition at post-intervention PA behavior + * PA behavior and leisure time exercise significantly increased within all conditions over time. PA behavior increased more for the approach imagery and approach-process imagery (= desired PS + action planning) conditions, compared to the process imagery and neutral control condition at T4 Mediator + * The effect of the approach imagery on PA behavior was significantly mediated by more action planning at T4 Quality assessment rating: 80%
Meijer (2018) [21] Mixed methods 2 × 3 factorial design Online The Netherlands	Smoking	*N*_baseline_ = 552 *N*_analysis_ = 339 *M*_age_ = 44.85 (SD = 17.39) 37.5% female	Smoking heaviness *M* = 15.71 (SD = 8.16) cigarettes daily Years smoked *M* = 26.75 (SD = 17.37)	Interventions Quitter self-identity PS intervention High social support vignette Active comparators PS intervention about washing hands No social support vignette Reading about heart and blood circulation (= control condition social support)	T0–baseline, followed by experimental manipulations T1–post-intervention T2–1-month follow-up T3–6-month follow-up Type of measurement Self-report Frequency Single session Duration Intervention: unclear Study: 6 months	Smoking-related identity + Participants in the strengthened quitter self-identity condition had marginally significantly stronger quitter self-identities at post-intervention than participants in the control condition (also when controlling for pre-test quit-intention) *Additional qualitative results* Almost half of participants (43%) made an explicit and positive link between quitting and their self-perception (e.g., quitting fits with self-perception as being determined, independent), and almost half of participants (48%) explicitly linked quitting to their lifestyle (e.g., having a healthy and conscious lifestyle). A small number of participants (9%) explicitly denied a quitter self-identity (e.g., ‘I am not someone who quits smoking’) or self-labeled as smoker (e.g., ‘I am a smoker’) Smoking precursor/behavior +—No significant differences in quit intention, changed smoking behavior or quit attempts between the strengthened quitter self-identity condition and the control condition at T2 or T3, when controlling for pre-test quitter self-identity Quality assessment rating: 67%
Meslot (2016) [56] Quantitative 2 × 2 factorial design Setting unclear UK	PA	*N*_baseline_ = 184 *N*_analysis_ = 176 (T1), 68 (T2) *M*_age_ = 26.95 (SD = 9.59) 52.7% female	PA frequency not reported	Interventions Mental simulation (= desired PS) Mental simulation + implementation intention Passive and active comparators Only completing study measures Implementation intention	T0–baseline T1–6-week follow-up T2–19-week follow-up (only participants from university campus fitness center) Type of measurement Objectively measured Frequency Single session Duration Intervention: unclear Study: ~ 5 months	PA behavior +—No statistically significant differences in fitness center attendance between conditions. No significant interaction between time and conditions Quality assessment rating: 40%
Murru (2010) [57] Quantitative RCT Online Canada	PA	*N*_baseline_ = 110 *N*_analysis_ = 80 (week 1–4) and 52 (week 1–8) *M*_age_ = 21.29 (SD = 3.23) 72.7% female	PA frequency < 3 bouts of at least 30 min of exercise a week	Interventions Desired PS intervention Undesired PS intervention Active comparator Quiz about PA	T0–baseline T1–post-intervention T2–4-week follow-up T3–8-week follow-up Type of measurement Self-report Frequency Single session Duration Intervention: unclear Study: 8 weeks	PA behavior + Participants in the PS conditions showed a greater increase in exercise minutes than the control condition, but this was not significant. When both PS conditions are combined, the difference becomes significant Moderator + The relationship between the combined PS conditions and exercise minutes during weeks 1–4 was reduced, indicating that planning self-efficacy partially mediated the effect of the combined PS conditions on exercise minutes. This was not significant, but the magnitude of the mediation effect indicated that 29% of the intervention effect was mediated by planning self-efficacy Quality assessment rating: 40%
Ouellette (2005) [17] Quantitative 2 × 2× 2 factorial design In person USA	PA	*N*_baseline_ = 197 *N*_analysis_ = 152 *M*_age_ = 20.00 (SD not reported) 60.5% female	PA frequency most participants reported exercising between 1–2 times a week	Interventions Desired PS intervention Undesired PS intervention Active comparators Imagine other person as exerciser Imagine other person as non-exerciser	T0–baseline T1–1-week follow-up T2–4-week follow-up Type of measurement Self-report Frequency Single session Duration: Intervention: unclear Study: 5 weeks	PA behavior +—No significant differences in exercise behavior between conditions (no statistics reported about the relevant effects) Moderator + * Consideration of Future Consequences (CFC) was a marginally significant moderator of the effect of the PS intervention on exercise behavior change over time. Participants high on CFC who were in the PS conditions showed a higher increase in exercise behavior from T1 to T2 compared to participants low on CFC in the PS conditions Unexpectedly, participants low on CFC who were in the control conditions also showed a higher increase in exercise behavior from T1 to T2 compared to participants high in CFC in the control conditions Quality assessment rating: 40%
Penfornis (2023) [20] Quantitative RCT Online The Netherlands	Smoking	*N*_baseline_ = 233 *N*_analysis_ = 157 (T2), 151 (T3) (used multiple imputation) *M*_age_ = 37.77 (SD = 18.57) 70.4% female	Smoking heaviness *M* = 12.10 (SD = 8.04) cigarettes daily Years smoked *M* = 20.23 (SD = 18.44)	Intervention Combined desired and undesired PS intervention Passive comparator Wait-list control	T0–baseline T1–post-intervention T2–1-month follow-up T3–3-month follow-up Type of measurement Self-report Frequency Single session (+ instruction to keep looking at the materials) Duration Intervention: unclear Study: 3 months	Smoking-related identity +—Smoker self-identity significantly decreased, and non-smoker self-identity significantly increased within all conditions over time. No within-condition effect for quitter self-identity, and no significant difference in the strength of smoker self-identity, quitter self-identity, or non-smoker self-identity between the two conditions at T1, T2, or T3 Quality assessment rating: 80%
Perras (2016) [58] Quantitative RCT Online Canada	PA	*N*_baseline_ = 294 *N*_analysis_ = 221 *M*_age_ = 63.40 (SD not reported) 68.5% female	PA frequency < 4 bouts of at least 15 min of MVPA a week	Interventions One-time desired PS intervention Repeated (= three times) desired PS intervention Passive comparator Only completing study measures	T0–baseline T1–4-week follow-up T2–8-week follow-up T3–12-week follow-up Type of measurement Self-report Frequency Single session or three sessions (1 per week) Duration Intervention: unclear Study: 12 weeks	PA-related identity +—PA identity significantly increased within all conditions over time, but there was no significant difference between conditions at T1, T2, or T3 PA behavior +—PA behavior significantly increased within all conditions over time, but there was no significant difference between conditions Quality assessment rating: 80%
Strachan (2017) [19] Quantitative RCT Online Canada	PA	*N*_baseline_ = 244 *N*_analysis_ = 198 *M*_age_ = 29.55 (SD = 10.84) 82.4% female	PA frequency *M* = 5.52 (SD = 4.93) bouts of at least 15 min of MVPA a week	Interventions Self-enhancing PS intervention (= desired PS) Self-regulatory PS intervention (= desired PS + action planning) Passive comparator Only completing study measures	T0–baseline T1–4-week follow-up T2–8-week follow-up Type of measurement Self-report Frequency Single session Duration Intervention: unclear Study: 8 weeks	PA behavior + * PA behavior increased significantly more in either of the two intervention conditions, compared to control. There was no significant difference between the intervention conditions Moderator + * Participants with higher task self-efficacy who were in the self-enhancing PS condition reported significantly higher levels of PA compared to those participants in the control condition at T1 and T2. Participants with lower task self-efficacy who were in the self-regulatory condition (= PS + action planning) reported significantly higher levels of PA compared to those participants in both the control condition and the self-enhancing condition at T1 Quality assessment rating: 80%
Multi-component interventions (*n* = 5)						
Cooke (2020) [59] Quantitative RCT In person Canada	PA	*N*_baseline_ = 145 *N*_analysis_ = 78 *M*_age_ = 37.20 (SD = 8.90) 100% female	PA frequency < 1 bout of exercise a week	Intervention PA intervention with exercise identity imagery (= desired PS) Active comparator PA intervention with imagery scripts with PA health information	T0–baseline T1–post-intervention (at week 9) T2–16-week follow-up T3–36-week follow-up Type of measurement Self-report Frequency 8 sessions (one per week) Duration Intervention: 3–5 min Study: 36 weeks	PA-related identity + * Exercise identity increased significantly more for the multi-component intervention condition compared to the control condition at post-intervention. However, effects were not sustained during follow-up PA behavior +—Exercise frequency significantly increased during week 2–4 and decreased in week 7. No significant main effect for condition and no condition x time interaction were found Quality assessment rating: 40%
Hollman (2022) [22] Mixed methods^d^ RCT In person Canada	PA	*N*_baseline_ = 41 *N*_analysis_ = 36 *M*_age_ = 38.39 (SD = 5.52) 100% female	PA frequency *M* = 70.73 min (SD = 61.25) of MVPA a week (insufficiently active^b^)	Intervention PA intervention with identity component Passive comparator Only completing study measures	T0–baseline T1–post-intervention Type of measurement Self-report Frequency 1 of the 10 weekly sessions was identity-related Duration Intervention: unclear Study: 10 weeks	PA-related identity + Exercise identity increased more strongly in the multi-component intervention condition compared to the control condition at post-intervention, but this was not significant PA precursor +—No statistically significant differences in decisional intention between conditions PA behavior + A greater percentage of participants in the multi-component intervention condition improved in PA behavior and met PA guidelines compared to the control condition. The difference was not significant, but the study was underpowered Quality assessment rating: 40%
Husband (2019) [60] Mixed methods^d^ RCT In person Canada	PA	*N*_baseline_ = 20 *N*_analysis_ = 18 *M*_age_ = 21.33 (SD = 2.30) 72.2% female	PA frequency Leisure Score Index GLTEQ: *M* = 26.14 (SD = 19.33) (sufficiently active)	Intervention Direct intervention (= PA intervention with identity component) Active comparator Indirect intervention (= PA intervention with no identity component)	T0–baseline T1–post-intervention (at 6 weeks) Type of measurement Self-report Frequency 3 sessions (bi-weekly) Duration Intervention: unclear Study: 6 weeks	PA-related identity - Large main effect of exercise identity within conditions over time. Exercise identity in the indirect intervention (= control) condition increased more than that in the direct intervention condition (= with identity component), but this effect had a small effect size (significance not reported) PA behavior + Large main effect of PA behavior within conditions over time. PA behavior in the direct intervention condition increased more than that in the indirect intervention (= control) condition, but this effect had a small effect size (significance not reported) Quality assessment rating: 40%
Morris (2019) [61] Quantitative 2 × 2 factorial design In person USA	Smoking	*N*_baseline_ = 256 *N*_analysis_ = 204 (T1), 165 (T2) (used multiple imputation) *M*_age_ = 18.92 (SD = 1.50) 45.3% female	Smoking frequency Occasional or daily Nicotine dependence FTND:* M* = 1.00 (SD = 1.41; range: 0–10) (very low dependence)	Interventions Unhealthy smoker prime (= imagine an unhealthy smoker of your age) Mortality prime (= describe emotions associated with own death) Active comparators Typical smoker prime (= imagine a typical smoker) Mortality control prime (= describe emotions associated with dental pain)	T0–baseline and post-intervention T1–3-week follow-up T2–6-week follow-up Type of measurement Self-report Frequency Single session Duration Intervention: unclear Study: 6 weeks	Smoking-related identity +—Smoker identity decreased over time in all conditions, but there was no significant difference between the unhealthy smoker prototype conditions and typical smoker prototype (= control) conditions Smoking precursor +—There was no significant difference in intention to quit between the unhealthy smoker prototype conditions and typical smoker prototype (= control) conditions Smoking behavior +—No significant main effect of smoker prototype on quit attempts or smoking frequency was found Quality assessment rating: 40%
Priebe (2020) and Wierts (2023) [49, 50] Mixed methods Pre-post design In person Canada	PA and smoking	Priebe (2020) *N*_baseline_ = 555 *N*_analysis_ = 216 (partly intention-to-treat) Most prevalent age group: 40–55 years 75.0% female Wierts (2023) *N*_analysis_ = 450 (used multiple imputation) 70.7% female	Priebe (2020) PA level *M* = 126.50 (SD = 159.02) min of MVPA (insufficiently active^b^) Smoking heaviness *M* = 11.40 cigarettes daily Years smoked *M* = 27.66 (SD = 11.48) Wierts (2023) PA level *M* = 67.77 (SD = 95.32) min of leisure time MVPA *M* = 56.55 (SD = 103.85) min of work/household-related MVPA (insufficiently active^b^) Smoking frequency 5.56% occasional 81.56% daily 9.78% not smoking	Intervention PA and smoking cessation intervention with desired PS component	T0–baseline (week 1) T1–post-intervention (week 10) Type of measurement Self-report and objectively measured Frequency Single session Duration Intervention: unclear Study: 10 weeks	PA-related identity + * Within subjects, runner identity significantly increased from baseline to end of the multi-component intervention Smoking-related identity + * Within subjects, smoker identity significantly decreased from baseline to end of the multi-component intervention PA behavior + * Within subjects, running frequency and MVPA significantly increased from baseline to end of the multi-component intervention Smoking behavior + * Within subjects, objective smoking behavior (CO scores) significantly decreased from baseline to end of the multi-component intervention Relationship between PA- and smoking-related identity and behavior + * Runner identity and smoker identity and their change scores were negatively correlated. As smoker identity decreased, runner identity increased. However, regression models show that running frequency is mainly predicted by runner identity (and not by smoker identity or the interaction between the identities), and quit rates are mainly predicted by smoker identity *Additional qualitative results* Qualitative findings were largely consistent with the quantitative results although more suggestive of an interaction between running and smoking identities. Participants described “seeing” themselves differently and often described themselves as either a smoker (or ex-smoker/non-smoker) or a runner, and sometimes transitioning from one to the other. Participants also mentioned running taking the focus away from quitting smoking Quality assessment rating: 74%
Possible-self avatar games (*n* = 4)						
Fox (2009 A) [51] *Study 2 in report* Quantitative 2 × 2 factorial design In person USA	PA	*N*_baseline_ = 60 *N*_analysis_ = 53 *M*_age_ = 20.54 (SD = 5.81) 39.6% female	Baseline PA level not measured	Interventions PS avatar + reward intervention (= exercising leads to avatar losing weight) PS avatar + punishment intervention (= inactivity leads to avatar gaining weight) Active comparators Generic avatar + reward intervention Generic avatar + punishment intervention	T0 – during intervention Type of measurement Objectively measured Frequency Single session Duration Intervention: unclear Study: unclear	PA behavior + * Participants exposed to the self-avatar performed significantly more exercise repetitions in the lab compared to the generic avatar Quality assessment rating: 40%
Fox (2009 B) [51] *Study 3 in report* Quantitative RCT In person USA	PA	*N*_baseline_ = 75 *N*_analysis_ = 73 *M*_age_ = 20.61 (SD = 2.50) 68.5% female	Baseline PA level not measured	Intervention Running PS avatar intervention Active comparators Running generic avatar intervention Inactive PS avatar intervention	T0–24 h after the intervention Type of measurement Self-report Frequency Single session Duration Intervention: unclear Study: unclear	PA behavior + * Participants observing an exercising (running) self-avatar exercised significantly more the day after the intervention compared to those in the exercising generic avatar condition and inactive self-avatar condition +—PA in the form of walking, climbing stairs, and biking (commuting) was also measured. No significant differences were found on these outcomes Quality assessment rating: 40%
Kastenmüller (2013) [62] Quantitative RCT In person UK	PA	*N*_analysis_ = 41 *M*_age_ = 20.88 (SD = 2.05) 41.5% female	Baseline PA level not measured	Intervention Jogging PS avatar intervention Active comparators Jogging generic avatar intervention Bowling generic avatar intervention	T0–post-intervention T1–1-week follow-up Type of measurement Self-report Frequency Single session Duration Intervention: unclear Study: 1 week	PA behavior + * Jogging with one’s own virtual character resulted in significantly more PA behavior in the week after the intervention than jogging or bowling with a generic virtual character Quality assessment rating: 40%
Song (2013) [63] Quantitative 2 × 2 factorial design In person USA	Smoking	*N*_baseline_ = 62 *N*_analysis_ = 62 *M*_age_ = 22.05 (SD = 3.28) 38.7% female	Smoking heaviness All participants smoked at least once in the last 30 days	Interventions Smoking cessation game with PS avatar + future condition (= aged face) Smoking cessation game with PS avatar + no future condition Active comparators Smoking cessation game with generic avatar + future condition Smoking cessation game with generic avatar + no future condition	T0–baseline T1–post-intervention Type of measurement Self-report Frequency Single session Duration Intervention: 10–15 min Study: unclear	Smoking precursor + Intention to quit smoking increased more in the self-avatar conditions compared to the generic avatar (= control) conditions, but this difference was not significant Limitation: identification with avatar was not significantly higher for the self- compared to the generic avatar condition, which suggests that the manipulation was not successful Quality assessment rating: 0%
Interventions challenging Identity (*n* = 3)						
Helweg-Larsen (2020) [52] *Study 1 in report* Quantitative RCT In person USA/ Denmark	Smoking	Danish sample *N*_baseline_ = 120 *N*_analysis_ = 111 *M*_age_ = 35.26 (SD = 16.60) 46.8% female American sample *N*_baseline_ = 120 *N*_analysis_ = 111 *M*_age_ = 39.41 (SD = 13.94) 43.2% female	Smoking heaviness Danish sample *M* = 15.83 (SD = 5.41) cigarettes daily American sample *M* = 18.10 (SD = 6.81) cigarettes daily	Intervention Stigma reminder about smoking Active comparator Stigma reminder about eczema	T0–pre-intervention T1–post-intervention Type of measurement Self-report Frequency Single session Duration Intervention: ~ 11 min Study: unclear	Smoking precursor + * Significant interaction of the smoking stigma intervention and smoking identity on smoking cessation intentions. When smoking identity was low, the smoking stigma condition (compared with the control condition) increased participants’ intentions to quit smoking, whereas there were no effects when smoking identity was medium or high Quality assessment rating: 40%
Helweg-Larsen (2020) [52] *Study 2 in report* Quantitative RCT In person USA	Smoking	*N*_baseline_ = 258 *N*_analysis_ = 194 *M*_age_ = 42.10 (SD = 12.02) 56.2% female	Smoking heaviness *M* = 20.5 (SD = 7.75) cigarettes daily	Intervention Stigma reminder about smoking Active comparator Stigma reminder about age	T0–pre-intervention T1–post-intervention Type of measurement Self-report Frequency Single session Duration Intervention: ~ 23 min Study: unclear	Smoking precursor +—There were no significant differences in smoking cessation intentions between the smoking stigma condition and the control condition Mediator/moderator + * Threat appraisal significantly mediated the effect of the smoking stigma intervention (i.e., participants exposed to the smoking stigma condition reported feeling more threatened than those in the control condition) on interest in smoking cessation tools +—Smoking identity did not moderate the mediational path from the smoking stigma intervention to smoking cessation intention through threat appraisals Quality assessment rating: 20%
Strachan (2008) [64] Quantitative Pre-post design Online Canada	PA	*N*_baseline_ = 165 *N*_analysis_ = 113 *M*_age_ = 32.89 (SD = 9.90) 70.0% female	Number of 30 + min bouts a week Mild PA *M* = 2.82 (SD = 2.64) Moderate PA *M* = 2.80 (SD = 2.06) Strenuous PA *M* = 3.37 (SD = 2.64)	Intervention Hypothetical exercise identity challenge vignette	T0–pre-intervention T1–post-intervention Type of measurement Self-report Frequency Single session Duration Intervention: unclear Study: unclear	PA precursor + * Those higher on exercise identity intended to exercise significantly more frequently and reported significantly stronger intentions to do so than those in the moderate identity condition during the hypothetical busier-than-usual 3 weeks Quality assessment rating: 80%

^a^The final MMAT quality assessment rating ranges from 0% (i.e., all biases rated as “no” or “can’t tell”; lowest rating) to 100% (i.e., all biases are rated as “yes”; highest rating), with 20% given for each bias scored as “yes”. For mixed-methods studies, for which 15 biases were evaluated instead of five, the total number of “yes” ratings was divided by three to calculate the overall rating.

^b^According to PA guidelines World Health Organization (WHO) [12]

^c^Frequency and intervention duration refers to the frequency and duration of the identity component

^d^Mixed-methods design, but only the quantitative results were relevant to the research aims of this systematic review. Therefore, the qualitative results were not reported

+ * = significant positive relationship; + = non-significant positive relationship; +—= non-significant unclear relationship;—= non-significant negative relationship; -* = significant negative relationship.

*PA* physical activity, *MMAT* Mixed Methods Appraisal Tool, *PS* possible-self, *Min* minutes, *RCT* randomized controlled trial, *CFC* consideration of future consequences, *MVPA* moderate-to-vigorous physical activity, *GLTEQ* Godin Leisure-Time Exercise Questionnaire [65], *FTND* Fagerstrom Test for Nicotine Dependence [66]

Across the 20 studies, the mean sample size included in the analysis (i.e., after attrition, if no missing data imputation was used) was 138 (range 18 [60]–339 [21]), totaling 2761 participants. For the studies reporting the mean age of participants (*n* = 18/20) [17, 19–22, 55–63], the average was 30.89 years (range mean 18.92 [61]–63.40 [58] years). Almost half of the studies (*n* = 8/20) [17, 56, 59–61, 63] focused on student samples, explaining the younger mean age. Approximately 64% of all participants were female, with two studies including females only [22, 59]. Of the studies focusing on PA (*n* = 14/20) [17, 19, 22, 50, 54–58, 60, 62, 63], one reported participants being sufficiently active (according to the Godin Leisure-Time Exercise Questionnaire [65]) [60] and three reported participants being insufficiently active at baseline (according to WHO guidelines [12]) [22, 50, 54]. For the remaining studies, the baseline PA level of participants was not reported (*n* = 4/14) [51AB, 55, 60] or it was unclear whether participants were sufficiently active or not (e.g., reported less than three bouts of at least 30 min of PA a week; *n* = 6/14) [17, 19, 57–59, 64]. The intervention of most PA studies focused on mild, moderate, or vigorous PA (*n* = 7/14) [19, 22, 57, 60, 62, 63], with other interventions focusing on exercise (i.e., structured PA chosen to do during one’s free time to enhance health or physical fitness [57]; *n* = 3/14) [17, 57, 59], running (*n* = 1/14) [50], walking in place (*n* = 1/14) [51A], fitness center attendance (*n* = 1/14) [56], or multiple types of PA (*n* = 1/14) [54]. Of smoking studies (*n* = 7/20) [20, 21, 50, 59, 61], all focused on individuals who smoked daily, on average 15.34 cigarettes (average based on *n* = 5/20) [20, 21, 50, 52AB], classified as moderate smokers [68, 69–70]. Other characteristics (e.g., socioeconomic position, BMI, smoking onset, nicotine dependence) were not summarized due to inconsistent reporting.

### Quality appraisal

Details on the quality assessment according to the MMAT guidelines [48] are reported in Additional file 4. The final quality assessment ratings for the included studies are also listed in the study characteristics table (see Table 1). For the studies with potentially high risks of bias (marked red in Additional file 4), it concerned the following biases: no blinding of the experimenter as to which condition participants were assigned to [56, 59, 60], unsuccessful manipulation (low identification with self-avatar in possible-self avatar game) [63], no clearly defined target population [49, 50], and a difference in exercise identity (outcome measure) between conditions at baseline (medium effect of *d* = 0.60) [60]. For all studies, the risk for one or multiple biases was unclear (marked orange in Additional file 4) due to poor reporting. For instance, randomization methods [17, 19–21, 56, 59–61] and whether conditions were comparable at baseline [17, 22, 55–57, 59–61] were both unclear for 12 of the 18 (67%) studies with controlled designs. Blinding of outcome assessors (i.e., the researchers or participants in case of self-report measurements) was also unclear for 11 of the 18 (61%) studies with controlled designs [17, 21, 22, 54, 59–61]. Additionally, none of the studies reported on blinding of outcome assessors performing the analyses, so this potential bias was not taken into account in the quality assessment, even though it could introduce a potential risk influencing outcomes [71].

### Identity-related interventions used in the context of PA promotion and smoking cessation

The first aim of this review was to describe the identity-related interventions used in the context of PA promotion and smoking cessation in individuals aged 12 years and over. We divided the interventions into four types (see Table 2 for an overview and descriptions). The most researched type is possible-self interventions (*n* = 8/20) [17, 19–21, 54, 56–58], in which participants imagine, think about, write about, and/or search for visuals related to their possible self to strengthen healthy identities and/or weaken unhealthy identities. The second type is multi-component interventions with an identity component (*n* = 5/20), which can consist of a possible-self intervention [50, 59] or other interventions to strengthen desired identities (e.g., positive self-talk related to being an exerciser) [22, 60, 61]. The third type is possible-self avatar games (*n* = 4/20), in which participants observe or control a virtual avatar representing a possible self [51AB, 60, 61]. The fourth type is interventions challenging identity (*n* = 3/20) [52AB, 62], in which participants are presented with a hypothetical identity challenge or stigmatization (e.g., situation in which participants experienced mistreatment due to their smoking) to examine reactions based on their PA- or smoking-related identity level. The identity-challenge intervention differs from the other three intervention types by focusing on the impact of an identity challenge rather than directly aiming to enhance healthy identities.

**Table 2:** Identity-related intervention types

Intervention type	Studies First author (year)	Description	Setting, mode of delivery, frequency, and duration	Behavior and identity	Additional information
Possible-self interventions *n* = 8	Chan (2012) [54] Meijer (2018) [21] Meslot (2016) [56] Murru (2010) [57] Ouellette (2005) [17] Penfornis (2023) [20] Perras (2016) [58] Strachan (2017) [19]	Participants imagine, think about, write about, and/or search for visuals related to their (un)desired possible self, which can be more proximal or distal in time (e.g., in 1 or 10 years). The aim is to strengthen healthy identities and/or weaken unhealthy identities	Setting In person: *n* = 2 Online: *n* = 5 Unclear: *n* = 1 Mode of delivery Via (online) written, audio or video instructions Frequency 1 session: *n* = 5 1 session and repeating by themselves: *n* = 2 1 vs. 3 sessions: *n* = 1	Behavior PA: *n* = 6 Smoking: *n* = 2 Identity construct Self-identity (Un)desired identity Desired:* n* = 5 Comparison both: *n* = 2 Combining both:* n* = 1 Identity labels A person who exercises regularly A person who is active Exerciser Physically active retiree A person who fails to exercise regularly Inactive person Non-smoker Quitter Smoker No specific label	**Example of instructions** “… We would like you to think about yourself in the future as a person who is a healthy, regular exerciser. … When you think about yourself 5 to 10 years from now as a healthy, regular exerciser, what images come to mind? Please take a few minutes to imagine and think about this image. After this, you will be asked to answer some questions about this image.”
Multi-component interventions *n* = 5	Cooke (2020) [59] Hollman (2022) [22] Husband (2019) [60] Morris (2019) [61] Priebe (2020) and Wierts (2023) [49, 50]	A multi-component intervention with an identity component as one of the BCTs The identity component consists of a possible-self intervention, imagining an unhealthy person who is a smoker of the same age (negative social framing of being a smoker), or bringing awareness to the concept of identity and strengthening feelings of the desired identity (e.g., positive self-talk related to being an exerciser)	Setting In person: *n* = 5 Frequency identity component 1 session: *n* = 2 Multiple sessions: *n* = 3 Duration study *M* = 13.6 weeks; 6–36 weeks	Behavior PA: *n* = 3 Smoking: *n* = 1 Both: *n* = 1 Identity construct Self-identity and role identity^a^ (Un)desired identity Desired:* n* = 4 Undesired: *n* = 1 Identity labels Exerciser Non-smoker Someone who quits smoking Smoker Unknown or no specific label	Examples of other BCTs^b^ in intervention PA - Information about health/emotional consequences (e.g., information on benefits of PA) - Information about others’ approval (e.g., imagery task on normative responses to PA engagement) - Problem solving (e.g., discuss injury prevention) - Prompts/cues (e.g., priming task on mortality) - Self-monitoring (with wearable) - Action planning (e.g., type of PA, sport participation, time) - Goal-setting Behavioral practice/rehearsal (i.e., supervised exercise training) Smoking - Problem solving (e.g., coaching on dealing with triggers and cravings) - Pros and cons (e.g., discuss pros and cons of quitting smoking) - Action planning (e.g., coaching on strategies for quitting) - Goal-setting (e.g., setting a quit date)
Possible-self avatar games *n* = 4	Fox (2009 A) [51] Fox (2009 B) [51] Kastenmüller (2013) [62] Song (2013) [63]	Participants observe and/or control a virtual avatar as a vivid visual representation of a possible self (intervention condition) compared to a generic/other avatar (control condition). Sometimes, additional manipulations such as showing a future avatar (aged face) versus a current avatar are included. Identification with the avatar is measured as a manipulation check	Setting In person: *n* = 4 Frequency 1 session: *n* = 4	Behavior PA: *n* = 3 Smoking: *n* = 1 Identity construct Self-identity (Un)desired identity Desired:* n* = 1 Undesired: *n* = 1 Comparison both: *n* = 1 Combining both:* n* = 1 Identity labels Smoker Social smoker Unknown or no specific label	Game elements PA - Recalling number sequences shown on the virtual avatar’s chest to ensure engagement with the avatar - Engaging in exercises to have the avatar mirror (in)activity Smoking - Jumping and running to avoid other smokers and cigarettes - Quiz or visualization of dangers and consequences of social smoking - Anticipated regret (e.g., game character expressing regret about not quitting smoking) - Presentation of facts about social smokers - Tips for quitting smoking
Interventions challenging identity *n* = 3	Helweg-Larsen (2020 A) [52] Helweg-Larsen (2020 B) [52] Strachan (2008) [64]	First, participants’ PA/smoking identity is measured. Second, participants read and/or give a short speech about a hypothetical identity challenge vignette or write about a specific situation in which they experienced mistreatment or discrimination because of their unhealthy behavior. Third, reactions to the vignette/writing task are compared between participants with stronger versus weaker PA/smoking identity. The aim is to explore reactions to identity challenges and compare these reactions between participants with different levels of PA- or smoking-related identities	Setting In person: *n* = 2 Online: *n* = 1 Frequency 1 session: *n* = 3	Behavior PA: *n* = 1 Smoking: *n* = 2 Identity construct Self-identity (Un)desired identity Challenging desired:* n* = 1 Challenging undesired: *n* = 2 Identity labels Exerciser Smoker	Example description vignette PA Participants are asked to imagine that due to work or school commitments, they have been much busier than usual over the past 3 weeks. The vignette suggests that this busy schedule has resulted in them being much less active than usual and this busy situation will continue for the next 3 weeks Smoking Participants are asked to read a newspaper article explaining that smoking is perceived as disgusting, negatively affects interpersonal and romantic relationships, and causes difficulty in getting and keeping a job

^a^Although some multi-component intervention studies focus on self-identity and others on role identity, both concepts are measured with the same questionnaire (the Exercise Identity Scale of Anderson and Cychosz, 1994) [67]. This indicates that, despite using different terms, these studies measure the same concept

^b^The behavior change techniques are categorized based on the behavior change technique taxonomy (v1) of Michie et al. (2013) [41]

*PA *physical activity, *BCTs* behavior change techniques

The most reported identity theories on which the identity-related interventions were based were PST [9] (*n* = 7/20) [17, 19–21, 57, 58, 63], identity theory [2, 72] (*n* = 5/20) [20, 50, 59, 60, 64], and social identity theories/models [72–75] (*n* = 3/20) [21, 50, 60]. None of the studies involved the target population in intervention development (i.e., co-creation), but five studies pilot-tested the intervention [17, 19, 20, 54, 64]. In most studies, participants completed the identity intervention only once (*n* = 14/20) [17, 19–21, 50, 55, 59–62], sometimes with instructions to repeat the exercise on their own (*n* = 2/20) [54, 57]. Only four studies repeated the identity intervention multiple times [22, 58–60]. All identity interventions focused on individual-level identity (i.e., self-identity or role identity) rather than interpersonal-level identity (e.g., social identity). For the three intervention types aiming to enhance healthy identities and behavior, most interventions focused on strengthening desired identities (*n* = 10/17) [19, 21, 22, 50, 54, 56, 58–60, 62], with two interventions focusing on weakening undesired identities [61, 63], one combining both approaches [20], and four comparing the two approaches [17, 56].

### Effects of identity-related interventions

The second aim of this review was to summarize the effects of identity-related interventions on PA- and smoking-related identities, and/or the promotion of PA and smoking cessation behaviors. The relevant results of the studies included in this review are summarized in Table 1. The results will be discussed per identity-related intervention type.

#### Possible-self interventions

Only three of the eight possible-self intervention studies measured the effect on PA- or smoking-related identities (see Table 1) [20, 21, 58]. One RCT investigated the effect of a possible-self intervention on *PA identity* and showed that PA identity changed significantly over time in all conditions, including the control group, but the changes did not significantly differ between conditions [58]. Similarly, for smoking, one RCT showed that both smoker self-identity and non-smoker self-identity significantly changed over time in all conditions, but no significant differences were found between conditions in the strength of *smoking-related identities* (i.e., smoker self-identity, quitter self-identity, or non-smoker self-identity) [20]. However, another factorial design study found that quitter self-identity (smoking) was marginally significantly stronger for the possible-self intervention condition compared to the control condition post-intervention [21].

Almost all possible-self intervention studies (*n* = 7/8) measured the effect on PA [17, 19, 54, 56–58] or smoking [21] behavior. Four of the six PA studies found no significant differences between the possible-self intervention condition(s) and the control condition(s) on *PA behavior* [17, 56–58]. Perras et al. [58] compared the effect of two intervention conditions—a single possible-self intervention session versus repeated possible-self intervention sessions—on PA-related identity and behavior, and found no significant differences between these conditions (and the control condition). This suggests that repeating a possible-self intervention multiple times does not enhance the effect on PA-related identity or PA behavior, though this conclusion is based on one study. Only one study [21] focused on the effect of a possible-self intervention on *smoking behavior.* Similar to four of the six PA studies, this factorial design study by Meijer et al. [21] found no significant differences between the possible-self intervention condition and the control condition on quit intention, changed smoking behavior, and quit attempts.

Two of the six possible-self intervention studies for PA found that PA intention and behavior increased significantly more in the possible-self intervention conditions compared to the control conditions [19, 54]. A possible explanation for why these two studies found positive significant effects, while the other four did not, could be due to pilot-testing the intervention. Both Chan and Cameron [54] and Strachan et al. [19] pilot-tested the possible-self intervention, which might have contributed to the effectiveness of the intervention. However, Ouellette et al. [17] also pilot-tested and did not find significant differences in PA behavior change between conditions. Another possible explanation might be the participants’ level of intention to increase PA at baseline. In Chan and Cameron [54], over 50% of participants in each intervention group were intending to change their behavior. Similarly, participants were only included in the study of Strachan et al. [19] if they had intentions to increase PA at baseline. This suggests that possible-self interventions for PA might be more effective for those better prepared to change their behavior. However, in the study of Murru and Ginis [57], only participants with an intention to exercise regularly were included, and no significant differences in PA behavior between conditions were found. The intention to increase PA at baseline was unclear for the other three possible-self intervention studies for PA [17, 56, 58]. Finally, the comparison of the intervention with different control conditions might also contribute to differences in results. For instance, Strachan et al. [19] compared the intervention with a passive comparator (i.e., participants in the control condition only completed study measures), possibly contributing to a significant difference in PA behavior change between groups. In contrast, most of the other studies used an active comparator (e.g., quiz about PA) [17, 57] or both [56]. However, Perras et al. [58] also included a passive comparator and did not find significant differences, while Chan and Cameron [54] included an active comparator and did find significant differences between conditions.

To conclude, the three possible-self intervention studies measuring the effect on PA- [58] or smoking-related identities [20, 21] suggest that possible-self interventions in their current form are not sufficient to change these identities. One smoking study [21] and four of the six PA studies [17, 56–58] found no significant differences between conditions in terms of smoking and PA behavior, while two PA studies did show significant positive effects [19, 54]. When comparing the different results, one could speculate that possible-self interventions for PA might be more suitable for those already prepared to change their behavior and that pilot testing the intervention could contribute to effectiveness. However, the mixed results and variation in study characteristics prevent firm conclusions.

#### Multi-component interventions

All five multi-component intervention studies investigated effects on identity, either PA- or smoking-related, and behavior, either PA or smoking (see Table 1) [22, 50, 59–61]. Most studies showed that following completion of the intervention, PA- [22, 50, 60] or smoking-related *identities * [50, 61] changed over time to healthier identities (within-subjects effect), though differences between conditions were often insignificant [22, 60, 61]. In the RCT by Hollman et al. [22], increases in PA identity were larger in the multi-component intervention condition compared to the control condition, but this difference was not significant. Moreover, in the RCT by Cooke et al. [59], exercise identity increased significantly more in the multi-component intervention condition at post-intervention compared to the control condition, but this effect was not sustained at the 16- and 36-week follow-ups. Conversely, in the RCT by Husband et al. [60], PA identity increased more in the control condition than in the intervention condition, but this effect had a small effect size (significance was not reported). However, both Husband et al. [60] and Hollman et al. [22] had very small sample sizes, respectively 18 and 36 participants included in the analysis, which could have contributed to the insignificant results.

A similar pattern was observed for the effect of multi-component interventions on *PA behavior*. All studies measuring PA behavior post-intervention reported a within-subjects increase in (short-term) PA behavior over time [22, 50, 59, 60]. In some studies, this increase was larger in the multi-component intervention condition compared to the control condition [22, 60]. However, none of these differences were significant. Likewise, the factorial design study by Morris et al. [61], focusing on *smoking*, found no significant differences in intention to quit smoking, quit attempts, or smoking frequency between conditions. Thus, while most studies show within-subject changes over time, the overall results are mixed, and the differences between conditions are often insignificant.

The mixed-methods pre-post study by Priebe et al. [50] was the only study included in this review to investigate an identity-related intervention targeting both PA promotion, specifically running, and smoking cessation. Results showed a significant increase in runner identity and behavior, and a significant decrease in smoker identity and objective smoking behavior from baseline to the end of the multi-component intervention. Moreover, the mixed-methods results indicated that although running frequency was mainly predicted by runner identity (and not by smoker identity or the interaction between the identities) and quit rates were mainly predicted by smoker identity [49], runner identity and smoker identity were shown to be negatively correlated [50]. These results suggest that it could be beneficial to target both PA promotion and smoking cessation in the same identity-related intervention.

In summary, multi-component intervention studies generally showed that PA- and smoking-related identities and behaviors shifted towards healthier identities and behaviors over time. However, none of the four controlled design studies found significant differences between conditions in PA or smoking behavior, possibly partly due to underpowered designs [22, 59–61]. One notable exception was the pre–post study by Priebe et al. [50], which successfully targeted both PA promotion and smoking cessation, suggesting the potential benefit of multi-behavior identity-related interventions.

#### Possible-self avatar games

None of the four possible-self avatar game studies measured PA- or smoking-related identity (see Table 1). The level of identification with the avatars was measured solely as a manipulation check. All three studies evaluating *PA behavior* reported a significant increase in PA behavior for the exercising self-avatar condition compared to the exercising other (i.e., generic) avatar condition [51AB, 60]. One of these studies measured the effect on PA behavior during the intervention in a lab setting [51A], while the other two studies measured PA behavior in daily life either the day [51B] or the week [62] after the intervention. These findings suggest that possible-self avatar games positively impact PA behavior not only in controlled settings but can also generalize to participants’ daily lives. However, it remains unclear whether possible-self avatar games also effectively promote *smoking cessation*, as only one study [63] investigated this, and the manipulation check in this study indicated that the manipulation was unsuccessful (i.e., identification with the self-avatar was not significantly higher than with the generic avatar).

In conclusion, while possible-self avatar games show promise for promoting PA behavior, their effects on smoking behavior remain unclear due to limited evidence.

#### Interventions challenging identity

In the three identity-challenge intervention studies [52AB, 62], participants were presented with hypothetical scenarios that challenged their PA or smoking identity (see Table 2). Unlike the other identity-related intervention types, these studies did not directly aim to promote healthier identities. Instead, they explored how individuals with different levels of PA- or smoking-related identities reacted when faced with an identity challenge. To investigate this, PA- or smoking-related identity was measured at baseline, and participants were divided by the researchers into groups with different identity levels (e.g., low versus high). This approach allowed for comparisons of how these groups responded to identity challenges.

In the pre–post study by Strachan and Brawley [64], the effect of a hypothetical exercise identity challenge (i.e., describing how a busy schedule made participants significantly less active than usual) on *PA intentions* was investigated in individuals with moderate and high exercise identity (see Table 1). They found that individuals with a higher exercise identity had stronger PA intentions when confronted with the exercise identity challenge compared to those with a moderate exercise identity. This finding supported their hypothesis that individuals with a high exercise identity respond to an identity challenge by seeking identity-behavior congruence [2]. Specifically, those with a high exercise identity experience negative consequences (e.g., emotions) due to the identity-behavior discrepancy, motivating them to reduce this discrepancy by intending to increase their PA.

For the two *smoking* identity challenge RCTs [52AB], the researchers hypothesized, based on the identity threat model of stigma [76], that challenging the identity of individuals who smoke would decrease their intentions to quit smoking. They also expected that individuals with a stronger smoking identity would react more strongly to an identity threat manipulation. Contrary to these expectations, Helweg-Larsen et al. [52] found in their first study that a hypothetical smoking identity challenge (i.e., presenting smoking as disgusting, and negatively affecting interpersonal relationships and employment opportunities) significantly increased *smoking cessation intentions*, but only among those with low smoking identity, not among those with medium or high smoking identity. In a second study, Helweg-Larsen et al. [52] found that a more personalized smoking identity challenge (i.e., writing about a situation in which participants experienced mistreatment or discrimination due to their smoking) increased interest in smoking cessation tools, but only when participants felt threatened by the identity challenge (i.e., mediation via threat appraisals). However, these results were not moderated by smoking identity.

Although the findings of the first study by Helweg-Larsen et al. [52] did not support their hypotheses, they are consistent with Strachan and Brawley’s results on PA intentions [64]. The results suggest that individuals with stronger healthy identities (high exercise identity) and weaker unhealthy identities (low smoking identity) might respond to identity challenges by intending to increase healthy behaviors (PA or smoking cessation behavior) to achieve identity–behavior congruency. However, this was not supported by the second study by Helweg-Larsen et al. [52], which did not find a moderating effect of smoking identity.

To conclude, although based on only three studies with varying identity-challenge formats, the results suggest that individuals with stronger healthy identities or weaker unhealthy identities may be more inclined to increase their intentions for healthy behaviors when faced with an identity challenge. However, it is important to note that these findings pertain to hypothetical identity challenges and their effects on behavioral precursors, such as intentions, rather than real-life identity challenges and actual changes in PA and smoking behavior.

### Mediators and moderators

The third aim of this review was to examine potential mediating or moderating factors of the effect of identity-related interventions on PA or smoking outcomes. However, only a few included studies investigated these factors using mediation or moderation analyses, and those that did only focused on possible-self interventions to promote PA (see Table 1) [17, 19, 54, 57].

#### Task and planning self-efficacy

Strachan et al. [19] found that *task self-efficacy* (i.e., the belief in one’s ability to organize and execute actions required to manage prospective situations) significantly moderated the effect of the possible-self intervention on *PA behavior*. Specifically, participants with higher task self-efficacy in the possible-self (i.e., self-enhancing) condition reported significantly higher PA levels at 4-week and 8-week follow-ups compared to participants with higher task self-efficacy in the control condition. Additionally, participants with lower task self-efficacy in the possible-self plus action planning (i.e., self-regulatory) condition showed significantly higher PA levels than participants with lower task self-efficacy in both the control and self-enhancing conditions at the 4-week follow-up. These findin gs suggest that possible-self interventions may be more effective in promoting PA behavior among individuals with higher task self-efficacy. They also indicate that adding an action planning component may enhance PA behavior in individuals with lower task self-efficacy. However, Murru and Ginis [57] found some evidence suggesting that *planning self-efficacy* (i.e., confidence in one’s ability to plan their exercise sessions) partially mediated the effect of the possible-self intervention conditions on *PA behavior*, though this result was not statistically significant. In contrast to Strachan et al. [19], the (non-significant) findings of Murru and Ginis [57] suggest that possible-self interventions alone (without an action planning component) might already boost self-efficacy for PA. Given that Strachan et al. [19] and Murru and Ginis [57] investigated different types of self-efficacy, it is possible that task self-efficacy moderates the effect of possible-self interventions on PA behavior, while planning self-efficacy mediates it.

#### Action planning

*Action planning* (i.e., specifying when, where, and how to act) [77, 78] was proposed as another potential mediator of the effect of possible-self interventions *on PA behavior* [54]. Although based on only one study, the results of Chan and Cameron [54] suggest that possible-self interventions might enhance individuals’ action planning, thereby increasing PA behavior. Comparing this with Murru and Ginnis’ [57] results, which suggested that planning self-efficacy might mediate the effect of possible-self interventions on PA (non-significantly), one could also infer that possible-self interventions might promote planning skills.

#### Consideration of future consequences

Another potential moderator of the effect of possible-self interventions on *PA behavior* is individuals’ *consideration of future consequences* (i.e., the extent to which individuals consider the immediate and distal consequences of their actions) [17, 58]. Ouellette et al. [17] found that participants with high consideration of future consequences in the possible-self condition showed a marginally significantly higher increase in exercise behavior compared to participants with low consideration of future consequences in the possible-self condition. Unexpectedly, participants with low consideration of future consequences in the control condition also showed a significantly higher increase in exercise behavior compared to participants with high consideration of future consequences in the control condition.

In summary, few of the included studies indicated that certain factors might mediate or moderate the effectiveness of identity-related interventions on PA behavior. Specifically, higher task self-efficacy and consideration of future consequences seem to enhance the impact of possible-self interventions on PA behavior. Additionally, possible-self interventions may influence PA behavior through mechanisms such as action planning and planning self-efficacy. However, these conclusions are based on a limited number of studies, and there is a lack of evidence regarding mediating or moderating factors for other identity-related interventions and on smoking outcomes.

## Discussion

This mixed-methods systematic review examined interventions directly targeting PA- and/or smoking-related identities to promote PA (i.e., a health-promoting behavior) and smoking cessation (i.e., a health-compromising behavior) in individuals aged 12 years and over. It also examined their effects on identity and behavior, as well as potential mediators or moderators of effectiveness. Across 20 studies, four types of identity-related interventions were identified: (1) possible-self interventions, in which participants imagine (un)desired possible selves to influence identity [17, 19–21, 54, 56–58], (2) multi-component interventions incorporating identity components [22, 50, 59–61], (3) possible-self avatar games, in which participants interact with a virtual representation of possible selves [51AB, 60, 61], and (4) identity-challenge interventions that assess the reactions of participants with different PA- or smoking-related identity levels to hypothetical identity challenges [52AB, 62]. Overall, intervention effectiveness was mixed: nearly half of the studies reported significant positive effects [19, 50, 54, 58, 60, 62], while the remaining studies found no significant differences between conditions [17, 20–22, 55–57, 59, 61, 63]. Importantly, no significant negative effects were reported. Results seem comparable for PA and smoking, although more research has focused on PA, complicating direct comparisons. All investigations into mediators and moderators involved possible-self interventions for PA. Included studies suggest that task self-efficacy [19] and consideration of future consequences [17] may moderate the effects of these interventions on PA behavior, while planning self-efficacy [57] and action planning [54] may mediate these effects. These findings highlight the role of self-regulatory strategies and future orientation in shaping behavior change outcomes.

Notably, the results for possible-self avatar games were in general more positive than those for the other three intervention types. Across the three possible-self avatar game studies focusing on PA, PA behavior increased significantly more in the intervention compared to the control condition [51AB, 60]. This suggests that vivid virtual representations (i.e., avatars) may be more effective in increasing PA behavior than imagination-based possible-self interventions, which depend on participants’ imaginative abilities [79, 80]. Evidence regarding smoking outcomes remains limited, as only one possible-self avatar game study investigated this with inconclusive results [63]. Future research could further explore the effectiveness of possible-self avatar games across various health behaviors and identify optimal operationalization strategies.

Only one study [50] investigated an identity-related intervention targeting both PA promotion and smoking cessation. Consistent with previous literature highlighting the benefits of targeting both behaviors [26–30], Priebe et al. [50] found that their intervention led to healthier identities and behaviors, suggesting that integrating PA and smoking cessation within a single intervention may be useful. Positive effects may also be attributed to the group-based format (e.g., group running sessions), which may have influenced social identity—a factor supported by previous empirical research as beneficial for health behavior change [1, 81]. As this was a pre-post study, more controlled research is needed. Furthermore, while our selection process allowed for the inclusion of studies on social identity, no eligible studies meeting all selection criteria were identified. In other words, all included studies focused merely on individual-level identity. Future research should explore the role of social identity in identity-related interventions.

In many included studies, identity and behavior improved over time (i.e., became healthier) across all conditions, but the differences between conditions were not always significant. This aligns with empirical research suggesting a reciprocal relationship between identity and behavior, where changes in one often drive changes in the other [5, 55, 82]. However, the exact causes of these changes remain unclear. One possibility is that study participation and self-monitoring of behavior might heighten awareness and prompt behavior change [58], a phenomenon known as the question-behavior effect [83]. Additionally, many included studies compared identity-related interventions with active comparators (e.g., imagery intervention focusing on action planning), which means that identity could be influenced both directly by the identity-related intervention and indirectly through behavior change techniques in the comparator intervention. To illustrate, Husband et al. [60] found that both health behavior change interventions with and without an identity component strengthened exercise identity over time. This highlights the need to investigate whether direct identity-related interventions offer additional value. A previous experimental study also suggested that identities and behaviors interact in a cyclical pattern [50]. Future research could further investigate this interaction to increase our knowledge on the optimal application of identity-related interventions, for instance, by measuring identity and behavior at multiple time points during and after interventions or conducting ecological momentary assessments.

Only a few included studies explored potential mediators and moderators of intervention effects, all of which concerned possible-self interventions for PA behavior. First, Strachan et al. [19] proposed that *task self-efficacy* moderated intervention effects. This is in contrast with previous reviews that indicate identity change may increase self-efficacy [1, 5], suggesting more of a mediating effect. Second, Murru and Ginis [57] and Chan and Cameron [54] found some indications that *planning self-efficacy* and *action planning* might mediate intervention effects. These findings align with the results of previous reviews [5, 6] and indicate that possible-self interventions may enhance self-regulatory strategies like planning [57], which in turn could support successful behavior change [5, 6, 77]. Finally, Ouellete et al. [17] found that *consideration of future consequences* moderated intervention effects on PA behavior. This factor might also influence PA- and smoking-related identities, as Perras et al. [58] and Penfornis et al. [20] found correlations with PA-related [58], quitter, non-smoker, and smoker self-identity [20]. Additionally, a previous meta-analysis [84] supports this role of consideration of future consequences in changing PA- or smoking-related identities and behavior. These findings underscore the importance of individual differences in tailoring possible-self interventions for behavior change. Future research should further investigate mediators and moderators for other identity-related intervention types and their effects on smoking behaviors.

None of the included studies examined demographic characteristics (e.g., age, socio-economic position) or behavior-specific characteristics (e.g., baseline PA, smoking onset age) as mediators or moderators. However, among the six studies on possible-self interventions for PA, only Chan and Cameron [54] and Strachan et al. [19] found significant positive results, and these studies focused mainly on participants already intending to increase their PA at baseline. This indicates that possible-self interventions may be more effective for individuals who are already inclined to increase PA. It is unclear if this also applies to smoking due to limited evidence. Future research should investigate who benefits most from identity-related interventions for PA and smoking, and how these interventions can support initiation, maintenance, and relapse recovery [6]. Understanding this will help tailor interventions to individual differences and behavior change phases.

### Strengths and limitations

To our knowledge, this is the first review synthesizing research on identity-related interventions for both PA and smoking cessation, enabling comparisons between the two. The review was pre-registered and followed a detailed protocol [31], employed a comprehensive three-step literature search strategy, and adhered to PRISMA guidelines [40]. The quality of the included studies was thoroughly assessed, with results presented in a detailed overview, including notes on the risk of biases. Additionally, no design or methodology restrictions were applied for study inclusion, ensuring a comprehensive overview of the literature on identity-related interventions aimed at directly influencing identity to promote PA and smoking cessation.

Despite its strengths, this review has several limitations. Some of these limitations are the result of methodological weaknesses of the studies included. For example, inconsistent reporting of behavior-specific baseline characteristics limits the generalizability of findings. Moreover, evidence for identity-challenge interventions and avatar games is based on only three and four studies respectively, and research on identity-related interventions for smoking is limited. Consequently, these results should be interpreted with caution. Additionally, all studies focused on individual-level identity (e.g., self-identity), while interpersonal-level identities (e.g., social identity) may also be important for behavior change [1, 81]. Also, while aiming to explore mediating or moderating effects of demographic, PA-, and smoking-specific characteristics, as noted in the review protocol [31], these effects were not investigated in the included studies. Furthermore, in the quality assessment, many biases could not be evaluated due to insufficient reporting. Future research should more clearly report sample characteristics and study procedures to facilitate risk of bias assessment, clarify the applicability of results to specific populations, and enhance the interpretation of findings. More research is also needed on identity-related interventions for smoking, identity-challenge interventions, avatar games, and interventions addressing social identity.

In addition, our review knows some methodological restrictions. First, we excluded studies that investigated interventions without a clear identity component. Our results, however, suggest that identity can also be indirectly influenced by other behavior change strategies. Future reviews could compare the effects of direct identity interventions and indirect behavior change interventions on identity. Second, the conceptualization of identity varies widely across studies, with overlapping but distinct terms (e.g., self-concept, integrated regulation) [5], which may have limited the sensitivity of our search strings to capture all relevant studies. We aimed to mitigate this by also performing backward and forward reference searches. Third, although we aimed for a mixed-methods review, the limited qualitative data resulted in a primarily quantitative focus. Fourth, the machine-learning screening software ASReview often deprioritizes records without abstracts, making them less likely to be screened by the reviewer and more likely to be marked as irrelevant. We mitigated this by independently double-screening 10% of the titles and abstracts and using additional search methods. Additionally, ASReview saves time and reduces the number of studies needing to be screened, allowing for more focused and accurate screening of the more relevant records. Fifth, 25% of the included studies were independently double-coded during data extraction, with discrepancies resolved through discussion. However, data extraction was not conducted in duplicate for all studies, which may have introduced some risk of extraction bias. Finally, while the MMAT’s broad criteria suit various study designs, they allow for more subjective interpretation. We addressed this by discussing criteria in detail before quality assessment, having one reviewer apply the MMAT consistently, double-coding a sample of articles by another reviewer, and discussing ambiguous cases within the research team.

### Recommendations

Based on our findings, several recommendations can be made for the design of future identity-related interventions in the context of PA and smoking. First, possible-self avatar games seem to be the most effective form of possible-self interventions for PA [51AB, 60]. These interventions not only show positive short-term effects in controlled settings [51A], but also seem to extend to participants’ daily PA behavior [51B, 60]. However, these conclusions are based on only three studies with college students, who may, for instance, be more receptive to avatar games than older adults. Second, imagination-based possible-self interventions for PA might be most effective for individuals already intending to increase PA [19, 54]. However, due to inconsistent reporting of baseline PA characteristics in many of the included studies, future research should investigate this further. Third, integrating PA promotion and smoking cessation into a single identity-related intervention could be beneficial, as suggested by one included study [50] and previous empirical studies [26–30]. More controlled studies are needed to explore this further. Fourth, the optimal design for different target populations remains unclear. For instance, it is still unclear whether identity interventions should be combined with other behavior change techniques, whether they should be repeated over time, and how to tailor them to different behavior change phases. Furthermore, factors like behavior change intention, planning skills [54, 57], self-efficacy [19], and consideration of future consequences [17, 20] are quite likely to influence the effectiveness of identity-related interventions. Moreover, identity labels and possible selves are suggested to be highly personal, varying in relevance between individuals [5]. Tailoring interventions to individual needs and skills could therefore enhance their effectiveness. While five studies in this review pilot-tested their intervention [17, 19, 20, 54, 64], none were developed in collaboration with their target population. Future research could incorporate pilot-testing and co-creation to better align with participant needs.

## Conclusions

This systematic review offers an overview of four types of identity-related interventions targeting PA or smoking. Our findings indicate that these interventions show potential, but questions remain about their optimal design and application for different populations. Although the results for PA and smoking seem similar, the limited research on smoking complicates direct comparisons. One study suggests that integrating PA promotion and smoking cessation in a single intervention could benefit individuals who can improve their lifestyle in both regards. Future research should further explore the mechanisms underlying identity-related interventions and whether these interventions target identity directly or indirectly through behavior change strategies, to better understand their optimal application. Additionally, since individual characteristics and skills (e.g., intention to change behavior, planning skills) seem to play a significant role, tailoring future interventions to their target population is important. Optimally designed identity-related interventions could serve as successful behavior change strategies, potentially reducing the negative consequences of low PA and smoking, and thereby contributing to public health.

## Supplementary Information

Additional file 1. PRISMA 2020 checklist.

Additional file 2. Screening manual for title and abstract and full-text screening.

Additional file 3. Search strings for electronic databases.

Additional file 4. Quality assessment according to the Mixed Methods Appraisal Tool guidelines.

## Data Availability

All data generated or analyzed during this study are included in this published article (and its additional files) or are available from the corresponding author upon reasonable request.

## Abbreviations

BMI: Body mass index
MMAT: Mixed Methods Appraisal Tool
OSF: Open Science Framework
PA: Physical activity
PRISMA: Preferred Reporting Items for Systematic Reviews and Meta-Analyses
PST: Possible Selves Theory
RCT: Randomized controlled trial
WHO: World Health Organization
