# Supplementary material for: The effect of identity-related interventions on physical activity- and smoking-related identities and behavior: a mixed-methods systematic review
Source: Syst Rev. 2026 Feb 24;15:106. doi: 10.1186/s13643-026-03103-2 (PMC13037006; doi:10.1186/s13643-026-03103-2)
Supplement: Supplementary file 2 — Additional file 2. Screening manual for title and abstract and full-text screening. [file 13643_2026_3103_MOESM2_ESM.docx]

**Additional file 2.** **Screening manual for title and abstract and full-text screening**

**Screening phase 1:** Title and Abstract (TiAb) screening (in ASReview and Rayyan)

|  | **Variable** | **Explanation** |
| --- | --- | --- |
| **ELIGIBILITY TiAb** | | |
| 1 | COMPLETENESS | There is an abstract available.  *Note*.  *If no abstract is available in the record management software, we will first search online for the abstract. If no abstract can be found but the article seems relevant based on the title, the record gets included.* |
| 2 | LANGUAGE | The title and abstract are written in a language spoken by the authors of the review (English, Dutch, French, or German) or in any language for which translation is available via Google Translate. |
| 3 | PEER-REVIEWED | The title and abstract belong to a peer-reviewed scientific publication.  *Note.*  *An exception is made for conference abstracts. Whenever a conference abstract meets all other criteria in this manual, it gets included.* |
| 4 | AGE | The record includes individuals aged 12 and over or the mean age of the sample is 12 years or higher.  *Note.*  *When this information is missing, the study is still eligible for full-text screening. The study is non-eligible when it is clear that the study includes participants younger than 12 years old (on average).* |
| 5 | BEHAVIOR |  |
|  | SMOKING        AND/OR PHYSICAL       ACTIVITY (PA) | The record focuses on (direct precursors of) smoking behaviors  ...  *Note*.  *Smoking behavior in this review refers to the act of smoking tobacco (any type) or electronic cigarettes. Smoking behaviors may encompass behaviors related to smoking initiation, maintenance and/or cessation.*    … and/or on (direct precursors of) recreational, non-professional PA behaviors - performed by the study participant(s) individually or together with a team they are part of - or on physical inactivity.  *Note.*  *PA behavior in this review refers to ‘bodily movements produced by* skeletal *muscles that require energy expenditure’ (WHO, 2018).*  *Records will be excluded when participants are structurally paid to engage in PA or sports. Records will be excluded when it is apparent that PA is performed in excess (e.g., as a result of an addiction to PA).* |
| 6 | SUBJECT |  |
|  | IDENTITY            IDENTITY INTERVENTIONS | The record studies smoking-related and/or PA-related self- or group-identity …    *Note.*  *Self-identity in this review refers to perceptions or views that people have of themselves.*  *Group identity in this review refers to perceptions or views that people derive from their membership to social groups.*  *In this review, self- or group-identity may encompass formation, maintenance and/or change.*  *The following concepts are known to differ from the definition of identity used in this review based on scientific definitions. Records focusing on one or more of these concepts will be considered as irrelevant and be excluded: self-esteem (but not collective self-esteem), self-efficacy, self-confidence, athletic identity, body image, body schema, self-compassion, body cathexis, perceived physical fitness, body (dis)satisfaction, self-acceptance, body representation, self-consciousness, subjective social status, self-forgiveness, group cohesion.*    … and/or the record studies identity-related intervention(s) or components of identity-related interventions aiming to influence smoking and/or PA behaviors and/or identities. |
| 7 | EXCLUSION | The record gets excluded when: |
|  |  | 7a – it does not relate to own smoking/PA or that of a group/team the study participant is part of (e.g., PA performed by a sports team the participant is a fan of, studying the identity of sport psychologists). |
|  |  | 7b – it **solely** examines identity concepts that have no relation to smoking and/or PA (e.g., changes in the general self-concept after a medical event) whatsoever. |
|  |  | 7c – a relation between smoking- and/or PA-related identity on one hand, and smoking and/or PA on the other hand is studied, but the relation is not a direct, mediation or moderation relation. |
|  |  | 7d – polysubstance are studied, and it is not possible to identify which findings pertain specifically to smoking. |
|  |  | 7e – the record aims to validate identity-related questionnaires/ methodologies/theories without investigating the direct relation between the questionnaire/methodologies/theories and smoking/PA behaviors. |

**Screening phase 2:** Full-text (FT) screening (in Excel)

|  | **Variable** | **Explanation** |
| --- | --- | --- |
| A | REFERENCE | Write down reference / title / author / DOI of record. |
| **ELIGIBILITY FT** | | |
| B | COMPLETENESS | There is a full-text available.  *Note*.  *If no full-text is accessible online, and if the record does not meet any exclusion criterium, we will proceed as follows:*   1. *The corresponding author of the record will be contacted to obtain a copy of the full record. In case of no reply, one reminder will be sent to obtain the full record and be excluded if it remains unavailable to this review team.* 2. *In case contact details of the corresponding author are outdated or unavailable, we will reach out to one other author of the record. In case of no reply, one reminder will be sent to the author to obtain the full record and be excluded if it remains unavailable to this review team.* |
| C | LANGUAGE | The full-text is written in a language spoken by the authors of the review (English, Dutch, French, or German) or in any language for which translation is available via Google Translate.  *Note.*  *Full-texts written in any other language than English, Dutch, French, or German will be assessed against below eligibility based on the translation from Google Translation. In case the translation is not deemed of sufficient quality to continue the screening process, the full-text will be excluded.* |
| D | PEER-REVIEWED | The full-text belongs to a peer-reviewed scientific publication.  *Notes.*  *a. An exception is made for conference abstracts for which we will proceed as follows:*   1. *Search for associated peer-reviewed published full-text online.* 2. *If full-text is available online, verify whether full-text is part of our dataset.* 3. *If full-text is part of our dataset → remove/exclude abstract.* 4. *If full-text is not part of our dataset → remove/exclude abstract, add full-text to our sample and screen full-text.* 5. *If no full-text is available, contact authors following the procedure in B.*   *b. Exclude dissertations and book (reviews). For commentaries, include when the commentary is about an eligible scientifically peer-reviewed article. If so, chart commentary together with the article.* |
| E | AGE | The record includes individuals aged 12 and over or the mean age of the sample is 12 years or higher. |
| F | INTERVENTION (COMPONENT) | The record studies an intervention aiming to influence smoking and/or PA behaviors and/or identities. |
| G | OUTCOME |  |
|  | SMOKING  AND/OR PA | The record focuses on (direct precursors of) smoking behaviors …  *Note*.  *Smoking behavior in this review refers to the act of smoking tobacco (any type) or electronic cigarettes. Smoking behaviors may encompass behaviors related to smoking initiation, maintenance and/or cessation.*  … and/or on (direct precursors of) recreational, non-professional PA behaviors - performed by the study participant(s) individually or together with a team they are part of - or on physical inactivity.  *Notes.*   1. *PA behavior in this review refers to ‘bodily movements produced by* skeletal *muscles that require energy expenditure’ (WHO, 2018).* 2. *Records will be excluded when participants are structurally compensated to engage in PA or sports. Records will also be excluded when it is apparent that PA is performed in excess (e.g., as a result of an addiction to PA).* |
|  | AND IDENTITY | Smoking-related and/or PA-related self- or group-identity are targeted by/measured as an outcome of the intervention.    *Notes.*   1. *Self-identity in this review refers to perceptions or views that people have of themselves.* 2. *Group identity in this review refers to perceptions or views that people derive from their membership to social groups.*   *In this review, self- or group-identity may encompass formation, maintenance and/or change.*   1. *The following concepts are known to differ from the definition of identity used in this review based on scientific definitions. Records focusing on one or more of these concepts will be considered as irrelevant and be excluded: self-esteem (but not collective self-esteem), self-efficacy, self-confidence, body image, body schema, self-compassion, body cathexis, perceived physical fitness, body (dis)satisfaction, self-acceptance, body representation, body self-perception(s), self-consciousness, subjective social status, self-forgiveness, group cohesion, physical self-presentation confidence, social identity unrelated to smoking or PA, physical self-concept (when meant as self-concepts about the physical/tangible self), global self-concept, self-construal, introjected regulation (Self-Determination Theory [SDT]), identified regulation (SDT), external regulation (SDT), intrinsic regulation (SDT), Prototype identification (Prototype Willingness Model), identity leadership,*   *Also see table below this full-text screening manual for an overview of questionnaires which are known to measure identity concepts that fall within or outside the scope of the review.* |
| H | EXCLUSION | The record gets excluded when: |
|  |  | Ha – it does not relate to own smoking/PA or that of a group/team the study participant is part of (e.g., PA performed by a sports team the participant is a fan of, studying the identity of sport psychologists). |
|  |  | Hb - it only observes spontaneously occurring smoking/PA behavior (changes) that are not initiated/stimulated by an intervention. |
|  |  | Hc – polysubstance is studied, and it is not possible to identify which findings pertain specifically to smoking. |
|  |  | Hd - a general smoking/PA intervention is researched that does not contain a clear identity component (e.g., aerobics classes). |
|  |  | He - the effect of a smoking/PA identity intervention is researched without a smoking-/PA-related identity outcome (e.g., exerciser identity or quitter identity) *OR* smoking/PA intention *OR* smoking/PA behavior outcome. |
|  |  | Hf - the smoking/PA multi-component intervention only contains a small identity component *AND* no smoking-/PA-related identity outcome was measured. |
|  |  | Hg - insufficient data or results were reported (only means but no statistical comparison; no specific data are presented for an identity component in a larger questionnaire, for example in the case of integrated regulation). |
|  |  | Hh - the record is a review (e.g., meta-analysis or systematic review) of prior scientific evidence.  *Note*. The review itself will be excluded from the final systematic review, but the reference list of the review will be manually searched for relevant records. Relevant records will be added to the dataset and screened against the above eligibility criteria. |
|  |  | Hi – the record is a research protocol or a study design paper, without results. |
| I | INCL-EXCL | Decision for inclusion or exclusion |
| J | NOTES | Notes / remarks |
| K | LINK | Optional: Full-text link |

**Questionnaires and related in-/exclusion criteria**

Below table illustrates the resulting screening decision whenever the listed questionnaires are employed to measure a self-concept related construct or being validated. This is a non-exhaustive list. The eligibility of questionnaires not listed in the table below will be assessed on a case-by-case basis.

| Exclude | Include |
| --- | --- |
| **Rosenberg self-esteem scale (Rosenberg, 1965)** does not have subscales (measures global self-esteem (Huang & Dong, 2012) | **Athletic identity measurement scale (AIMS) (Brewer, Van Raatle, & Linder, 1993)**  Measures athletic identity (the extent to which one identifies as an athlete) which we decided to exclude in the definition table. Include when it refers to an individual identifying with being a non-professional athlete. Otherwise exclude. |
| **Body-esteem scale (BSE, Mendelson, Mendelson & White, 2001)** subscales: Body Esteem Appearance, Body Esteem Weight, and Body Esteem Attribution (i.e., evaluations attributed to others about one’s body/appearance) |  |
| **Multidimensional self-esteem test** (subscales: Interpersonal relationships, environmental control competence, emotionality, schooling achievement, family life, bodily experience, global self-esteem, Tremolada et al., 2017) | **Academic and Athletic Identity Scale (AAIS) (Yukhymenko–Lescroart, 2014)**  Measures academic and athletic identity. Same comment as above. |
| **(Children’s) perceived (physical) competence scale (Harter, 1982)** subscales: cognitive competence, social competence, physical competence | **Tennessee self-concept scale (TSCS)** (subscales for ‘internal’ are identity, self-satisfaction and behavior. Subscales for ‘external’ are physical self, moral ethical-self, personal self, family self and social self) → INCLUDE if internal scale (identity) is measured |
| **Heatherton and Polivy State Self-esteem Scale (Heatherton & Polivy, 1991)** subscales: performance self-esteem, social self-esteem, appearance self-esteem) |  |
|  | **Tennessee scale of self-image**  subscales: negative self-concept (self-criticism)  positive self-concept (physical self, moral-ethical self, personality self, family self, social self, identity, self-image, behaviour, self-evaluation) (Faganel & Tušak, 2005)  → INCLUDE if identity scale relates to smoking and/or PA |
| **Body Cathexis Scale (BCS) factors:** subordinate and independent body features; physical health and fitness; sexual; face and overall appearance; weight; physique/strength; health and effectiveness (Hammond & Rourke, 1983) |  |
| **Baller Identity Measurement Scale (Harrison et al., 2010)** (subscales: social identity, exclusivity, positive- and negative affectivity)  Measures identification with being a ‘baller’ which is used to refer to profiles recruited for revenue sports. Although it does include relevant ideas ‘I consider myself a baller’, ‘Balling is the most important part of my life’, | Behavioral Regulation in Exercise Questionnaire (Wilson, Rodgers, Loit & Scime, 2006) including the integrated regulation subscale (but NOT BREQ-2) subscales: External, Introjected, Identified, Integrated intrinsic (the last one being the one we want and has also been shown to be valid on its own)  Includes items which are linked to exercise identity such as “I consider exercise to be part of my identity” and “I consider exercise a fundamental part of who I am” |
| **Harter Self-perception Profile for Children (Granleese et al., 1994)** (subscales: Scholastic competence, Social acceptability, Athletic competence, Physical appearance, Behavioral conduct, Global self-worth) |  |
|  |  |
| **Social physique anxiety scale**  considering we exclude articles on physique anxiety (see definition table) |  |
| **Richard's Physical self - concept scale (1987)** (subscales: body build, action, appearance, health, physical ability, strength and satisfaction) |  |
| **Pictorial scale of physical self-concept (Estevan et al., 2019)** (Subscales: Sportiness, Flexibility, Endurance, Speed, Strength, Coordination, Appearance, Enjoyment) |  |
| **Situational Motivation Scale (SIMS)** (Subscales: Intrinsic motivation, Identified motivation, External regulation and Amotivation) |  |
| **Piers-Harris Self-concept scale** (subscales: behavior, intellectual and school status, physical appearance and attributes, anxiety, popularity, happiness and satisfaction) (Piers & Harzberg, 2002) |  |
| **Self-perception profile** (subscales: bodily attractiveness, sports competence, physical strength, physical conditioning, general physical self-worth) |  |
| **Children and Youth Physical Self Perception Profile (CY-PSPP; Eklund, Whitehead, & Welk, 1997)** (subscales: sport/athletic competence, attractive body adequacy, strength competence, condition/stamina competence, global physical self-worth and global self-worth) |  |
| **Physical Self-Concept questionnaire** (subscales: ability, condition, attractiveness and strength) |  |
| **(Physical) self-description questionnaire** (designed to measure 11 scales: Strength, Body Fat, Activity, Endurance/Fitness, Sports Competence, Coordination, Health, Appearance, Flexibility, Global Physical Self-concept, and Global Esteem) (Marsch, 1995) (Marsh & O'Niell, 1984)  Fox (1998): ‘PSDQ provides a comprehensive assessment of self-perceptions covering a wide range of subdomains of the physical self (p. 306)’. |  |
| **Self-concept form-5 (AF-5) subscales:** academic self-concept, social self-concept, emotional self-concept, family self-concept, physical self-concept (Valero et al., 2020) |  |
| **Self-concept clinical inventory (SCCI) subscales:** social acceptance/rejection, self-efficacy, psychological maturity, impulsiveness (Pombo et al., 2016) |  |
| **Physical self-perception profile (PSPP) (Fox & Corbin, 1989)** subscales: Sport, Condition, Body, Strength, Physical self-worth |  |
| **Physical self-efficacy scale**  subscales: Perceived Physical Ability, Physical Self-Presentation Confidence (Ryckman et al., 1982) |  |
| **Physical self-inventory**  subscales: general self-esteem, physical self-worth, sport competence, physical condition, attractive body, physical strength (Ninot et al., 2001) |  |
| **Multidimensional Self Concept Scale (MSCS) (Bracken, 1992)**  subscales: social, competence, affect, academic, family, and physical self-concept (Rotatori, 1994) |  |
| **Treatment Self-Regulation Questionnaire (TSRQ) (Ryan & Connell, 1989)**  Subscales: Autonomous motivation (no item about identity), Introjected regulation, External regulation, Amotivation |  |
| Behavioral Regulation in Exercise Questionnaire-2 (BREQ-2) (Markland & Tobin, 2004) Subscales: Amotivation, external‚ introjected‚ identified and intrinsic motivation  Only item being slightly relevant: “It is important to me to exercise regularly” (BREQ and BREQ-2) part of the identified regulation subscale |  |
| The Exercise Motivations Inventory - 2 (EMI-2) (Markland & Hardy, 1993) |  |
| Identity style inventory, Revised (ISI3) (Berzonsky et al. (2013): Stylistic differences in how people approach or attempt to evade the task of forming, maintaining, and/or revising their sense of identity. —> So more focused on a general identity concept (and not PA/smoking identity specifically) |  |
| Motives for Physical Activities Measure – Revised (MPAM-R): list of reasons why people engage in physical activities, sports and exercise, including intrinsic reasons. → Does not include any item relating to identity |  |
