# Supplementary material for: The effect of identity-related interventions on physical activity- and smoking-related identities and behavior: a mixed-methods systematic review
Source: Syst Rev. 2026 Feb 24;15:106. doi: 10.1186/s13643-026-03103-2 (PMC13037006; doi:10.1186/s13643-026-03103-2)
Supplement: Supplementary file 3 — Additional file 3. Search strings for electronic databases. [file 13643_2026_3103_MOESM3_ESM.docx]

**Additional file 3. Search strings for electronic databases**

**PubMed**

<http://www.ncbi.nlm.nih.gov/pubmed?otool=leiden>

Excl. Book chapters, dissertations and commentaries

(("Identification, Psychological"[majr] OR "Social Identification"[majr] OR "identity"[ti] OR "Self Concept"[majr:noexp] OR "Self Concept"[ti] OR "self perception*"[ti] OR "self image*"[ti] OR "self view*"[ti] OR "sense of self"[ti] OR "sense of identity"[ti] OR "sense of identities"[ti] OR "Social Identification"[majr] OR "Social Identification"[ti] OR "self identity"[ti] OR "self identities"[ti] OR "social identity"[ti] OR "social identities"[ti] OR "identity formation*"[ti] OR "identity maintenance"[ti] OR "identity change*"[ti] OR "identity process*"[ti] OR "identity related intervention*"[ti] OR "self conception"[ti] OR "self conceptions"[ti] OR ("identit*"[ti] AND ("construct*"[ti] OR "chang*"[ti] OR "creat*"[ti])) OR "self schema"[ti] OR "self schemata"[ti] OR "self schema*"[ti] OR "selfschema"[ti] OR "selfschemata"[ti] OR "selfschema*"[ti] OR "self definition"[ti] OR "self defin*"[ti] OR "selfdefinition"[ti] OR "selfdefin*"[ti] OR "possible self"[ti] OR "possible selves"[ti] OR "possible self*"[ti] OR "possible selv*"[ti] OR "future self"[ti] OR "future selves"[ti] OR "future self*"[ti] OR "future selve*"[ti] OR "prototype self"[ti] OR "prototype selves"[ti] OR "prototype self*"[ti] OR "prototype selv*"[ti]) AND ("Smoking"[majr] OR "Smoking"[ti] OR "Smoking Cessation"[majr] OR "Smoking Devices"[majr] OR "cigar"[ti] OR "cigars"[ti] OR "cigarette"[ti] OR "cigarettes"[ti] OR "tobacco"[ti] OR "smoker"[ti] OR "smoker*"[ti] OR "smokers"[ti] OR "smoking behavior*"[ti] OR "smoking behaviour*"[ti] OR "tobacco use cessation*"[ti] OR "nicotine use cessation"[ti] OR "quitting smoking*"[ti] OR "quit smoking*"[ti] OR "stop smoking*"[ti] OR "smoking reduction"[ti] OR "smoking abstinence"[ti] OR "cessation"[ti] OR "Exercise"[majr] OR "Exercise"[ti] OR "Exercis*"[ti] OR "Physical Activity"[ti] OR "Physical Activit*"[ti] OR "Physically Activ*"[ti] OR "Physical Inactivit*"[ti] OR "Physically Inactiv*"[ti] OR "physical exercise*"[ti] OR "Sports"[majr] OR "sports"[ti] OR "sport"[ti] OR "Physical Fitness"[majr] OR "Athletic Performance"[majr] OR "fitness"[ti] OR "physical training"[ti] OR "athletic activity"[ti] OR "Athletic Performance"[ti]) NOT ("Animals"[mesh] NOT "Humans"[mesh]) NOT ("Comment"[Publication Type] OR "Editorial"[Publication Type] OR "Comment"[ti] OR "Editorial"[ti]))

**Web of Science**

<http://isiknowledge.com/wos>

UT=(000558534900023 OR 000243668000016 OR 000436535300005 OR 000523837700005)

TI=(("Psychological Identification" OR "Social Identification" OR "identity" OR "Self Concept" OR "Self Concept" OR "self perception*" OR "self image*" OR "self view*" OR "sense of self" OR "sense of identity" OR "sense of identities" OR "Social Identification" OR "Social Identification" OR "self identity" OR "self identities" OR "social identity" OR "social identities" OR "identity formation*" OR "identity maintenance" OR "identity change*" OR "identity process*" OR "identity related intervention*" OR "self conception" OR "self conceptions" OR (identit* AND (construct* OR chang* OR creat*)) OR "self schema" OR "self schemata" OR "self schema*" OR "selfschema" OR "selfschemata" OR "selfschema*" OR "self definition" OR "self defin*" OR "selfdefinition" OR "selfdefin*" OR "possible self" OR "possible selves" OR "possible self*" OR "possible selv*" OR "future self" OR "future selves" OR "future self*" OR "future selve*" OR "prototype self" OR "prototype selves" OR "prototype self*" OR "prototype selv*") AND ("Smoking" OR "Smoking" OR "Smoking Cessation" OR "Smoking Devices" OR "cigar" OR "cigars" OR "cigarette" OR "cigarettes" OR "tobacco" OR "smoker" OR "smoker*" OR "smokers" OR "smoking behavior*" OR "smoking behaviour*" OR "tobacco use cessation*" OR "nicotine use cessation" OR "quitting smoking*" OR "quit smoking*" OR "stop smoking*" OR "smoking reduction" OR "smoking abstinence" OR "cessation" OR "Exercise" OR "Exercise" OR "Exercis*" OR "Physical Activity" OR "Physical Activit*" OR "Physically Activ*" OR "Physical Inactivit*" OR "Physically Inactiv*" OR "physical exercise*" OR "Sports" OR "sports" OR "sport" OR "Physical Fitness" OR "Athletic Performance" OR "fitness" OR "physical training" OR "athletic activity" OR "Athletic Performance" OR "health behavior*" OR "health behaviour*") NOT ("veterinary" OR "rabbit" OR "rabbits" OR "animal" OR "animals" OR "mouse" OR "mice" OR "rodent" OR "rodents" OR "rat" OR "rats" OR "pig" OR "pigs" OR "porcine" OR "horse" OR "horses" OR "equine" OR "cow" OR "cows" OR "bovine" OR "goat" OR "goats" OR "sheep" OR "ovine" OR "canine" OR "dog" OR "dogs" OR "feline" OR "cat" OR "cats")) NOT (DT=("Editorial Material" OR "Book Review" OR "News Item" OR "Book Chapters") OR TI=("Editorial" OR "Book Review" OR "Comment") OR AK=("Editorial" OR "Book Review" OR "Comment"))

**PsycINFO**

<http://search.ebscohost.com/login.aspx?authtype=ip,uid&profile=lumc&defaultdb=psyh>

an(2007-05710-001)

(TI(("Psychological Identification" OR "Social Identification" OR "identity" OR "Self Concept" OR "Self Concept" OR "self perception*" OR "self image*" OR "self view*" OR "sense of self" OR "sense of identity" OR "sense of identities" OR "Social Identification" OR "Social Identification" OR "self identity" OR "self identities" OR "social identity" OR "social identities" OR "identity formation*" OR "identity maintenance" OR "identity change*" OR "identity process*" OR "identity related intervention*" OR "self conception" OR "self conceptions" OR (identit* AND (construct* OR chang* OR creat*)) OR "self schema" OR "self schemata" OR "self schema*" OR "selfschema" OR "selfschemata" OR "selfschema*" OR "self definition" OR "self defin*" OR "selfdefinition" OR "selfdefin*" OR "possible self" OR "possible selves" OR "possible self*" OR "possible selv*" OR "future self" OR "future selves" OR "future self*" OR "future selve*" OR "prototype self" OR "prototype selves" OR "prototype self*" OR "prototype selv*") AND ("Smoking" OR "Smoking" OR "Smoking Cessation" OR "Smoking Devices" OR "cigar" OR "cigars" OR "cigarette" OR "cigarettes" OR "tobacco" OR "smoker" OR "smoker*" OR "smokers" OR "smoking behavior*" OR "smoking behaviour*" OR "tobacco use cessation*" OR "nicotine use cessation" OR "quitting smoking*" OR "quit smoking*" OR "stop smoking*" OR "smoking reduction" OR "smoking abstinence" OR "cessation" OR "Exercise" OR "Exercise" OR "Exercis*" OR "Physical Activity" OR "Physical Activit*" OR "Physically Activ*" OR "Physical Inactivit*" OR "Physically Inactiv*" OR "physical exercise*" OR "Sports" OR "sports" OR "sport" OR "Physical Fitness" OR "Athletic Performance" OR "fitness" OR "physical training" OR "athletic activity" OR "Athletic Performance" OR "health behavior*" OR "health behaviour*") NOT ("veterinary" OR "rabbit" OR "rabbits" OR "animal" OR "animals" OR "mouse" OR "mice" OR "rodent" OR "rodents" OR "rat" OR "rats" OR "pig" OR "pigs" OR "porcine" OR "horse" OR "horses" OR "equine" OR "cow" OR "cows" OR "bovine" OR "goat" OR "goats" OR "sheep" OR "ovine" OR "canine" OR "dog" OR "dogs" OR "feline" OR "cat" OR "cats")) **OR** ((MJ "Social Identity" OR MJ "Group Identity" OR MJ "Professional Identity" OR MJ "Identity Formation" OR MJ "Self-Concept") AND (MJ "Tobacco Smoking" OR MJ "Electronic Cigarettes" OR MJ "Passive Smoking" OR MJ "Smokeless Tobacco" OR MJ "Physical Activity" OR MJ "Actigraphy" OR MJ "Exercise" OR MJ "Sports" OR MJ "Athletes" OR MJ "Baseball" OR MJ "Basketball" OR MJ "Extreme Sports" OR MJ "Football" OR MJ "Judo" OR MJ "Martial Arts" OR MJ "Professional Sports" OR MJ "Soccer" OR MJ "Swimming" OR MJ "Tennis" OR MJ "Weightlifting") NOT ("veterinary" OR "rabbit" OR "rabbits" OR "animal" OR "animals" OR "mouse" OR "mice" OR "rodent" OR "rodents" OR "rat" OR "rats" OR "pig" OR "pigs" OR "porcine" OR "horse" OR "horses" OR "equine" OR "cow" OR "cows" OR "bovine" OR "goat" OR "goats" OR "sheep" OR "ovine" OR "canine" OR "dog" OR "dogs" OR "feline" OR "cat" OR "cats"))**)** NOT (PT("book" OR "Dissertation Abstract") OR TI("Editorial" OR "Book Review" OR "Comment"))

**Cochrane**

<https://www.cochranelibrary.com/advanced-search/search-manager>

(("Psychological Identification" OR "Social Identification" OR "identity" OR "Self Concept" OR "Self Concept" OR "self perception*" OR "self image*" OR "self view*" OR "sense of self" OR "sense of identity" OR "sense of identities" OR "Social Identification" OR "Social Identification" OR "self identity" OR "self identities" OR "social identity" OR "social identities" OR "identity formation*" OR "identity maintenance" OR "identity change*" OR "identity process*" OR "identity related intervention*" OR "self conception" OR "self conceptions" OR ((identity OR identities) AND (constructing OR change OR create)) OR "self schema" OR "self schemata" OR "self schema*" OR "selfschema" OR "selfschemata" OR "selfschema*" OR "self definition" OR "self defin*" OR "selfdefinition" OR "selfdefin*" OR "possible self" OR "possible selves" OR "possible self*" OR "possible selv*" OR "future self" OR "future selves" OR "future self*" OR "future selve*" OR "prototype self" OR "prototype selves" OR "prototype self*" OR "prototype selv*") AND ("Smoking" OR "Smoking" OR "Smoking Cessation" OR "Smoking Devices" OR "cigar" OR "cigars" OR "cigarette" OR "cigarettes" OR "tobacco" OR "smoker" OR "smoker*" OR "smokers" OR "smoking behavior*" OR "smoking behaviour*" OR "tobacco use cessation*" OR "nicotine use cessation" OR "quitting smoking*" OR "quit smoking*" OR "stop smoking*" OR "smoking reduction" OR "smoking abstinence" OR "cessation" OR "Exercise" OR "Exercise" OR "Exercis*" OR "Physical Activity" OR "Physical Activit*" OR "Physically Activ*" OR "Physical Inactivit*" OR "Physically Inactiv*" OR "physical exercise*" OR "Sports" OR "sports" OR "sport" OR "Physical Fitness" OR "Athletic Performance" OR "fitness" OR "physical training" OR "athletic activity" OR "Athletic Performance" OR "health behavior*" OR "health behaviour*")):ti

**Embase**

<http://ovidsp.ovid.com/ovidweb.cgi?T=JS&PAGE=main&MODE=ovid&D=oemezd>

((*"Identity"/ OR "identity".ti OR "Self Concept".ti OR "self perception*".ti OR "self image*".ti OR "self view*".ti OR "sense of self".ti OR "sense of identity".ti OR "sense of identities".ti OR "Social Identification".ti OR "self identity".ti OR "self identities".ti OR "social identity".ti OR "social identities".ti OR "identity formation*".ti OR "identity maintenance".ti OR "identity change*".ti OR "identity process*".ti OR "identity related intervention*".ti OR "self conception".ti OR "self conceptions".ti OR ("identit*".ti AND ("construct*".ti OR "chang*".ti OR "creat*".ti)) OR "self schema".ti OR "self schemata".ti OR "self schema*".ti OR "selfschema".ti OR "selfschemata".ti OR "selfschema*".ti OR "self definition".ti OR "self defin*".ti OR "selfdefinition".ti OR "selfdefin*".ti OR "possible self".ti OR "possible selves".ti OR "possible self*".ti OR "possible selv*".ti OR "future self".ti OR "future selves".ti OR "future self*".ti OR "future selve*".ti OR "prototype self".ti OR "prototype selves".ti OR "prototype self*".ti OR "prototype selv*".ti) AND (exp *"Smoking"/ OR "Smoking".ti OR *"Smoking Cessation"/ OR exp *"Smoking Device"/ OR "cigar".ti OR "cigars".ti OR "cigarette".ti OR "cigarettes".ti OR "tobacco".ti OR "smoker".ti OR "smoker*".ti OR "smokers".ti OR "smoking behavior*".ti OR "smoking behaviour*".ti OR "tobacco use cessation*".ti OR "nicotine use cessation".ti OR "quitting smoking*".ti OR "quit smoking*".ti OR "stop smoking*".ti OR "smoking reduction".ti OR "smoking abstinence".ti OR "cessation".ti OR exp *"Exercise"/ OR "Exercise".ti OR "Exercis*".ti OR "Physical Activity".ti OR "Physical Activit*".ti OR "Physically Activ*".ti OR "Physical Inactivit*".ti OR "Physically Inactiv*".ti OR "physical exercise*".ti OR exp *"Sport"/ OR "sports".ti OR "sport".ti OR *"Fitness"/ OR "fitness".ti OR "physical training".ti OR "athletic activity".ti OR "Athletic Performance".ti) NOT (exp "Animals"/ NOT exp "Humans"/) NOT ("editorial"/ OR "Comment".ti OR "Editorial".ti OR "book"/))

**Emcare** <http://ovidsp.ovid.com/ovidweb.cgi?T=JS&NEWS=n&CSC=Y&PAGE=main&D=emcr>

((*"Identity"/ OR "identity".ti OR "Self Concept".ti OR "self perception*".ti OR "self image*".ti OR "self view*".ti OR "sense of self".ti OR "sense of identity".ti OR "sense of identities".ti OR "Social Identification".ti OR "self identity".ti OR "self identities".ti OR "social identity".ti OR "social identities".ti OR "identity formation*".ti OR "identity maintenance".ti OR "identity change*".ti OR "identity process*".ti OR "identity related intervention*".ti OR "self conception".ti OR "self conceptions".ti OR ("identit*".ti AND ("construct*".ti OR "chang*".ti OR "creat*".ti)) OR "self schema".ti OR "self schemata".ti OR "self schema*".ti OR "selfschema".ti OR "selfschemata".ti OR "selfschema*".ti OR "self definition".ti OR "self defin*".ti OR "selfdefinition".ti OR "selfdefin*".ti OR "possible self".ti OR "possible selves".ti OR "possible self*".ti OR "possible selv*".ti OR "future self".ti OR "future selves".ti OR "future self*".ti OR "future selve*".ti OR "prototype self".ti OR "prototype selves".ti OR "prototype self*".ti OR "prototype selv*".ti) AND (exp *"Smoking"/ OR "Smoking".ti OR *"Smoking Cessation"/ OR exp *"Smoking Device"/ OR "cigar".ti OR "cigars".ti OR "cigarette".ti OR "cigarettes".ti OR "tobacco".ti OR "smoker".ti OR "smoker*".ti OR "smokers".ti OR "smoking behavior*".ti OR "smoking behaviour*".ti OR "tobacco use cessation*".ti OR "nicotine use cessation".ti OR "quitting smoking*".ti OR "quit smoking*".ti OR "stop smoking*".ti OR "smoking reduction".ti OR "smoking abstinence".ti OR "cessation".ti OR exp *"Exercise"/ OR "Exercise".ti OR "Exercis*".ti OR "Physical Activity".ti OR "Physical Activit*".ti OR "Physically Activ*".ti OR "Physical Inactivit*".ti OR "Physically Inactiv*".ti OR "physical exercise*".ti OR exp *"Sport"/ OR "sports".ti OR "sport".ti OR *"Fitness"/ OR "fitness".ti OR "physical training".ti OR "athletic activity".ti OR "Athletic Performance".ti) NOT (exp "Animals"/ NOT exp "Humans"/) NOT ("editorial"/ OR "Comment".ti OR "Editorial".ti OR "book"/))

**PsycArticles**

Via PsycINFO

TI(("Psychological Identification" OR "Social Identification" OR "identity" OR "Self Concept" OR "Self Concept" OR "self perception*" OR "self image*" OR "self view*" OR "sense of self" OR "sense of identity" OR "sense of identities" OR "Social Identification" OR "Social Identification" OR "self identity" OR "self identities" OR "social identity" OR "social identities" OR "identity formation*" OR "identity maintenance" OR "identity change*" OR "identity process*" OR "identity related intervention*" OR "self conception" OR "self conceptions" OR (identit* AND (construct* OR chang* OR creat*)) OR "self schema" OR "self schemata" OR "self schema*" OR "selfschema" OR "selfschemata" OR "selfschema*" OR "self definition" OR "self defin*" OR "selfdefinition" OR "selfdefin*" OR "possible self" OR "possible selves" OR "possible self*" OR "possible selv*" OR "future self" OR "future selves" OR "future self*" OR "future selve*" OR "prototype self" OR "prototype selves" OR "prototype self*" OR "prototype selv*") AND ("Smoking" OR "Smoking" OR "Smoking Cessation" OR "Smoking Devices" OR "cigar" OR "cigars" OR "cigarette" OR "cigarettes" OR "tobacco" OR "smoker" OR "smoker*" OR "smokers" OR "smoking behavior*" OR "smoking behaviour*" OR "tobacco use cessation*" OR "nicotine use cessation" OR "quitting smoking*" OR "quit smoking*" OR "stop smoking*" OR "smoking reduction" OR "smoking abstinence" OR "cessation" OR "Exercise" OR "Exercise" OR "Exercis*" OR "Physical Activity" OR "Physical Activit*" OR "Physically Activ*" OR "Physical Inactivit*" OR "Physically Inactiv*" OR "physical exercise*" OR "Sports" OR "sports" OR "sport" OR "Physical Fitness" OR "Athletic Performance" OR "fitness" OR "physical training" OR "athletic activity" OR "Athletic Performance" OR "health behavior*" OR "health behaviour*") NOT ("veterinary" OR "rabbit" OR "rabbits" OR "animal" OR "animals" OR "mouse" OR "mice" OR "rodent" OR "rodents" OR "rat" OR "rats" OR "pig" OR "pigs" OR "porcine" OR "horse" OR "horses" OR "equine" OR "cow" OR "cows" OR "bovine" OR "goat" OR "goats" OR "sheep" OR "ovine" OR "canine" OR "dog" OR "dogs" OR "feline" OR "cat" OR "cats"))

**Psychology and Behavioral Sciences Collection**

Via PsycINFO

Excluding Book Reviews

TI(("Psychological Identification" OR "Social Identification" OR "identity" OR "Self Concept" OR "Self Concept" OR "self perception*" OR "self image*" OR "self view*" OR "sense of self" OR "sense of identity" OR "sense of identities" OR "Social Identification" OR "Social Identification" OR "self identity" OR "self identities" OR "social identity" OR "social identities" OR "identity formation*" OR "identity maintenance" OR "identity change*" OR "identity process*" OR "identity related intervention*" OR "self conception" OR "self conceptions" OR (identit* AND (construct* OR chang* OR creat*)) OR "self schema" OR "self schemata" OR "self schema*" OR "selfschema" OR "selfschemata" OR "selfschema*" OR "self definition" OR "self defin*" OR "selfdefinition" OR "selfdefin*" OR "possible self" OR "possible selves" OR "possible self*" OR "possible selv*" OR "future self" OR "future selves" OR "future self*" OR "future selve*" OR "prototype self" OR "prototype selves" OR "prototype self*" OR "prototype selv*") AND ("Smoking" OR "Smoking" OR "Smoking Cessation" OR "Smoking Devices" OR "cigar" OR "cigars" OR "cigarette" OR "cigarettes" OR "tobacco" OR "smoker" OR "smoker*" OR "smokers" OR "smoking behavior*" OR "smoking behaviour*" OR "tobacco use cessation*" OR "nicotine use cessation" OR "quitting smoking*" OR "quit smoking*" OR "stop smoking*" OR "smoking reduction" OR "smoking abstinence" OR "cessation" OR "Exercise" OR "Exercise" OR "Exercis*" OR "Physical Activity" OR "Physical Activit*" OR "Physically Activ*" OR "Physical Inactivit*" OR "Physically Inactiv*" OR "physical exercise*" OR "Sports" OR "sports" OR "sport" OR "Physical Fitness" OR "Athletic Performance" OR "fitness" OR "physical training" OR "athletic activity" OR "Athletic Performance" OR "health behavior*" OR "health behaviour*") NOT ("veterinary" OR "rabbit" OR "rabbits" OR "animal" OR "animals" OR "mouse" OR "mice" OR "rodent" OR "rodents" OR "rat" OR "rats" OR "pig" OR "pigs" OR "porcine" OR "horse" OR "horses" OR "equine" OR "cow" OR "cows" OR "bovine" OR "goat" OR "goats" OR "sheep" OR "ovine" OR "canine" OR "dog" OR "dogs" OR "feline" OR "cat" OR "cats"))

**Academic Search Premier**

<http://search.ebscohost.com/login.aspx?authtype=ip,uid&profile=lumc&defaultdb=aph>

TI(("Psychological Identification" OR "Social Identification" OR "identity" OR "Self Concept" OR "Self Concept" OR "self perception*" OR "self image*" OR "self view*" OR "sense of self" OR "sense of identity" OR "sense of identities" OR "Social Identification" OR "Social Identification" OR "self identity" OR "self identities" OR "social identity" OR "social identities" OR "identity formation*" OR "identity maintenance" OR "identity change*" OR "identity process*" OR "identity related intervention*" OR "self conception" OR "self conceptions" OR (identit* AND (construct* OR chang* OR creat*)) OR "self schema" OR "self schemata" OR "self schema*" OR "selfschema" OR "selfschemata" OR "selfschema*" OR "self definition" OR "self defin*" OR "selfdefinition" OR "selfdefin*" OR "possible self" OR "possible selves" OR "possible self*" OR "possible selv*" OR "future self" OR "future selves" OR "future self*" OR "future selve*" OR "prototype self" OR "prototype selves" OR "prototype self*" OR "prototype selv*") AND ("Smoking" OR "Smoking" OR "Smoking Cessation" OR "Smoking Devices" OR "cigar" OR "cigars" OR "cigarette" OR "cigarettes" OR "tobacco" OR "smoker" OR "smoker*" OR "smokers" OR "smoking behavior*" OR "smoking behaviour*" OR "tobacco use cessation*" OR "nicotine use cessation" OR "quitting smoking*" OR "quit smoking*" OR "stop smoking*" OR "smoking reduction" OR "smoking abstinence" OR "cessation" OR "Exercise" OR "Exercise" OR "Exercis*" OR "Physical Activity" OR "Physical Activit*" OR "Physically Activ*" OR "Physical Inactivit*" OR "Physically Inactiv*" OR "physical exercise*" OR "Sports" OR "sports" OR "sport" OR "Physical Fitness" OR "Athletic Performance" OR "fitness" OR "physical training" OR "athletic activity" OR "Athletic Performance" OR "health behavior*" OR "health behaviour*") NOT ("veterinary" OR "rabbit" OR "rabbits" OR "animal" OR "animals" OR "mouse" OR "mice" OR "rodent" OR "rodents" OR "rat" OR "rats" OR "pig" OR "pigs" OR "porcine" OR "horse" OR "horses" OR "equine" OR "cow" OR "cows" OR "bovine" OR "goat" OR "goats" OR "sheep" OR "ovine" OR "canine" OR "dog" OR "dogs" OR "feline" OR "cat" OR "cats"))
