# Supplementary material for: The effect of identity-related interventions on physical activity- and smoking-related identities and behavior: a mixed-methods systematic review
Source: Syst Rev. 2026 Feb 24;15:106. doi: 10.1186/s13643-026-03103-2 (PMC13037006; doi:10.1186/s13643-026-03103-2)
Supplement: Supplementary file 4 — Additional file 4. Quality assessment according to the Mixed Methods Appraisal Tool guidelines. [file 13643_2026_3103_MOESM4_ESM.docx]

| **Additional file 4. Quality assessment according to the Mixed Methods Appraisal Tool guidelines** | | | | | | |
| --- | --- | --- | --- | --- | --- | --- |
| **Table S4.1.**  Mixed Methods Appraisal Tool (MMAT) assessment for quantitative studies with randomized controlled and factorial designs (*n* = 17/20) | | | | | | |
| **Article:** First author (year) | **Biases**^a^ |  |  | |  | **Notes**  Overall quality assessment rating^b^ |
|  | **Appropriate randomiza-tion** | **Groups comparable at baseline** | **Complete outcome assessment** | **Blinded outcome assessment**^c^ | **Participants’ adherence** |  |
| **Possible-self interventions** (*n* = 7) | | | | | | |
| Chan (2012) |  |  |  |  |  | **Randomization:** A randomization check was performed using a Latin square design to assign group sessions to conditions.  **Groups comparable**: No significant baseline differences between conditions.  **Outcome assessment:** 76% of the sample was retained throughout the study. Dropouts did not significantly differ from completers in terms of demographic variables or baseline levels of relevant outcome measures. Also, intention-to-treat analyses were conducted. **Blindnes**s: Participants were blinded, but it is unclear whether the experimenter was blinded. As the study was conducted in person, this could have influenced the results**.** **Adherence**: The intervention was pilot-tested and adherence was monitored through diaries and follow-up phone calls. Adherence checks indicated that adherence was adequate, with no significant differences between conditions.  **Overall quality assessment rating:** 80% |
| Meslot (2016) |  |  |  |  |  | **Randomization:** Participants were randomized using a random numbers table.  **Groups comparable:** Significant differences between conditions regarding gender, but not reported whether this affected the results. No other variables showed significant differences.  **Outcome assessment:** Approximately 95% of the sample was retained at T2, but there was a significant drop-out at T3 (63%). Dropouts differed significantly from completers in baseline fitness center attendance and perceived behavioral control, but the authors controlled for this in the analyses. Also, missing data were imputed using multiple imputation. **Blindness:** Participants were blinded to group assignment, but the experimenter was not. **Adherence:** Adherence assessment was not reported.  **Other:** There were inconsistencies in reporting follow-up timepoints and sample sizes. This was clarified once the authors were contacted, the correct Ns and follow-up timepoints are reported in Table 1.  **Overall quality assessment rating:** 40% |
| Murru (2010) |  |  |  |  |  | **Randomization:** Used method not specified.  **Groups comparable:** Not reported.  **Outcome assessment:** Daily reminders were sent to participants to fill in logbooks with outcome assessments. However, significant dropout from T1-T2 (33%) and T2-T3 (57%), resulting in small group sizes at the post-intervention comparisons. It was not reported whether drop-outs differed significantly from completers.  **Blindness:** Not reported. As the study was conducted online, there was no direct contact between participants and experimenter. **Adherence:** A manipulation check was performed, and adherence was adequate.  **Other:**  - Results remained robust when controlling for baseline exercise.  - Design was adjusted (conditions collapsed) after reviewing the data; therefore, analyses should be considered exploratory, and conclusions should be limited.  **Overall quality assessment rating:** 40% |
| Ouellette (2005) |  |  |  |  |  | **Randomization**: Used method not specified.  **Groups comparable**: Not reported.  **Outcome assessment**: Significant dropout from T2-T3 (33%). There were no significant differences in baseline and relevant outcome variables between completers and drop-outs.  **Blindness**: Unclear whether participants and the experimenter were blinded. As the study was conducted in person, this could have influenced the results**.** **Adherence**: The intervention was pilot-tested and manipulation checks were conducted.  **Other:**  - Authors conclude that the interventions affected behavior. However, based on the results, the intervention does not seem to significantly affect behavior.  **Overall quality assessment rating:** 40% |
| Penfornis (2023) |  |  |  |  |  | **Randomization:** Used method not specified.  **Groups comparable:** No significant baseline differences between conditions  **Outcome assessment**: Higher dropout rate in the experimental group than in the control group, but no significant differences in background characteristics between dropouts and completers. Missing data were estimated using maximum likelihood estimation and robustness checks were conducted. **Blindness:** Not reported. As the study was conducted online, there was no direct contact between participants and the experimenter. **Adherence:** The intervention was pilot-tested and compliance checks were conducted. 28 participants (12%) were categorized as non-compliers to at least one visual or verbal possible-self task.  **Overall quality assessment rating:** 80% |
| Perras (2016) |  |  |  |  |  | **Randomization:** Participants were randomized using a single multiple-choice question with nonsensical answer choices. Each answer choice was linked to a different group assignment and the choices were randomized with each page view.  **Groups comparable:** The significance of baseline differences between conditions was not reported.  **Outcome assessment:** Drop-out was not related to demographic variables or condition. In the “repeated” possible-self condition, follow-up tasks completion was satisfactory, with 89% completing the second task and 73% completing the third task. **Blindness**: Not reported. As the study was conducted online, there was no direct contact between participants and the experimenter. **Adherence**: Manipulation and compliance checks were conducted, resulting in the removal of 16 participants (approximately 5%) due to nonadherence/ineligibility.  **Overall quality assessment rating:** 80% |
| Strachan (2017) |  |  |  |  |  | **Randomization**: Used method not specified.  **Groups comparable**: No significant baseline differences between conditions, except for marital status, which did not affect the results.  **Outcome assessment**: Most participants completed all time points (>80%) and there were no significant differences between dropouts and completers. **Blindness:** Not reported. As the study was conducted online, there was no direct contact between participants and the experimenter. **Adherence**: The intervention was pilot-tested and manipulation checks were conducted, confirming successful manipulation.  **Overall quality assessment rating:** 80% |
| **Multi-component interventions** (*n* = 4) | | | | | | |
| Cooke (2020) |  |  |  |  |  | **Randomization:** Participants were randomized using a computer-generated list of random numbers.  **Groups comparable:** No significant baseline differences between conditions.  **Outcome assessment:** Only 53% of the sample remained at the last follow-up. Dropouts were slightly younger than completers (authors did not correct for this), but there were no differences in other demographic variables or baseline levels of relevant outcome measures.  **Blindness:** The experimenter was not blinded. As the study was conducted in person, this could have influenced the results**. Adherence:** Not reported whether adherence was assessed.  **Other:** The authors do not clearly specify how they arrived at the final values for exercise behavior (whether they used the logbook only or also the Godin Leisure-Time Exercise Questionnaire).  **Overall quality assessment rating:** 40% |
| Hollman (2022)^c^ |  |  |  |  |  | **Randomization:**  Participants were randomized using “Block Randomization in Clinical Trials” in Microsoft Excel.  **Groups comparable:** The significance of baseline differences between conditions was not reported.  **Outcome assessment:** Five (12%) participants dropped out. The authors did not report if dropouts differed from completers. **Blindness:** Unclear whether participants and the experimenter were blinded. As the study was conducted in person, this could have influenced the results**. Adherence:** Adherence assessment was not reported.  **Other:** This was a feasibility study with a small sample size, which was underpowered to detect significant differences in outcomes relevant for this study.  **Overall quality assessment rating:** 40% |
| Husband (2019)^c^ |  |  |  |  |  | **Randomization:** Participants were randomized using a random number generator.  **Groups comparable:** The indirect group (control condition) had higher exercise identity (relevant outcome measure) at baseline than the direct group (multi-component intervention with identity component), with a medium effect size of *d* = .60.  **Outcome assessment:** 90% of participants completed the study (only two dropouts). The authors did not report if dropouts differed from completers. **Blindness:** The experimenter was not blinded. As the study was conducted in person, this could have influenced the results. **Adherence:** One-on-one sessions were conducted, but adherence outside of the supervised sessions was not monitored.  **Other:** On average participants did not meet the inclusion criterion of insufficient activity (they were, on average, sufficiently active).  **Overall quality assessment rating:** 40% |
| Morris (2019) |  |  |  |  |  | **Randomization:** Used method not specified**.**  **Groups comparable:** Not reported.  **Outcome assessment:** Significant dropout at T1 (21%) and T2 (36%). There were no significant differences between dropouts and completers and authors used missing data imputation. **Blindness:** Unclear whether participants and the experimenter were blinded. As the study was conducted in person, this could have influenced the results**.** **Adherence:** A manipulation check was conducted.  **Other:** Authors were contacted regarding a clarification question about the results but could not provide further information.  **Overall quality assessment rating:** 40% |
| **Avatar games** (*n* = 4) | | | | | | |
| Fox (2009 A) |  |  |  |  |  | **Randomization:** Used method not specified.  **Groups comparable:** Not reported.  **Outcome assessment**: There was minimal dropout (12%). **Blindness**: Unclear whether participants and the experimenter were blinded. As the study was conducted in person, this could have influenced the results**.** **Adherence**: A manipulation check was conducted.  **Overall quality assessment rating:** 40% |
| Fox (2009 B) |  |  |  |  |  | **Randomization:** Used method not specified.  **Groups comparable:** Not reported.  **Outcome assessment:** There was minimal dropout (3%). **Blindness**: Unclear whether participants and the experimenter were blinded. As the study was conducted in person, this could have influenced the results**.** **Adherence:** A manipulation check was conducted.  **Other:**  - Authors checked whether participants correctly identified the study’s purpose (none did, rated by the researcher and an independent blind rater).  - Main author was contacted about a question regarding the results but did not respond.  **Overall quality assessment rating:** 40% |
| Kastenmüller (2013) |  |  |  |  |  | **Randomization:** Used method not specified.  **Groups comparable**: Not reported.  **Outcome assessment**: Only one outlier participant (2%) was removed from the analysis (> 3 standard deviations over the mean in the outcome variable). **Blindness**: Unclear whether participants and the experimenter were blinded. As the study was conducted in person, this could have influenced the results**.** **Adherence**: A manipulation check was conducted.  **Overall quality assessment rating:** 40% |
| Song (2013) |  |  |  |  |  | **Randomization:** Used method not specified.  **Groups comparable:** Gender was balanced across conditions. Other baseline differences between groups were not reported.  **Outcome assessment:** No information on attrition or missing data. **Blindness**: Unclear whether participants and the experimenter were blinded. As the study was conducted in person, this could have influenced the results**.** **Adherence**: A manipulation check was conducted. Identification with avatar was not significantly higher for the self- compared to the generic avatar condition, suggesting that the manipulation was not successful.  **Overall quality assessment rating:** 0% |
| **Interventions challenging identity** (*n* = 2) | | | | | | |
| Helweg-Larsen (2020 A) |  |  |  |  |  | **Randomization:** Used method not specified.  **Groups comparable**: Not reported.  **Outcome assessment**: 18 participants (8%) were excluded after data collection due to personal experience with the control condition topic (eczema), failure to meet eligibility criteria, or inability or unwillingness to fully participate in the study. No participants dropped out in response to the manipulation. **Blindness**: Unclear whether participants and the experimenter were blinded. As the study was conducted in person, this could have influenced the results**.** **Adherence**: To ensure engagement with the identity challenge vignette, participants were asked to give a speech about it, which was video recorded.  **Other:** Main author was contacted about a question regarding the procedure and responded with a clarification.  **Overall quality assessment rating:** 40% |
| Helweg-Larsen (2020 B) |  |  |  |  |  | **Randomization:** Used method not specified.  **Groups comparable**: Not reported.  **Outcome assessment**: 50 participants dropped out of the study, and data from 14 additional participants (totaling 25%) were removed because they did not follow the intervention instructions.  **Blindness**: Unclear whether participants and the experimenter were blinded. As the study was conducted in person, this could have influenced the results**.** **Adherence**: Adherence checks were conducted, and if participants who did not adhere to the manipulation were removed from the study.  **Overall quality assessment rating:** 20% |

Legend: Green = yes (no indication of bias); orange = can’t tell/no information was given; red = no (indication of bias)

*Notes*.

^a^More information about the biases and criteria can be found in the MMAT manual [1].

^b^The final MMAT quality assessment rating ranges from 0% (i.e., all biases rated as ‘no’ or ‘can’t tell’; lowest rating) to 100% (i.e., all biases are rated as ‘yes’; highest rating), with 20% given for each bias scored as ‘yes’.

^c^Blinded outcome assessment only refers to whether researchers were blinded to intervention assignment during data collection. None of the articles mentioned whether the analysis was blinded or not.

^d^Mixed-methods design, but only the quantitative results were relevant for the research aims of this systematic review. Therefore, the qualitative results were not taken into account.

**Table S4.2.**

MMAT assessment for mixed-methods studies (*n* = 2/20).

| **Article:** First author (year) | **Biases**^a^ |  |  |  |  | **Notes**  Overall quality assessment rating^b^ |
| --- | --- | --- | --- | --- | --- | --- |
| **Possible-self interventions** (*n* = 1) | | | | | | |
| Meijer (2018) | **QUANTITATIVE** (randomized controlled) | | | | |  |
|  | **Appropriate randomiza-tion** | **Groups comparable at baseline** | **Complete outcome assessment** | **Blinded outcome assessment**^c^ | **Participants’ adherence** |  |
|  |  |  |  |  |  | **Randomization:** Used method not specified.  **Groups comparable**: Marginally significant differences in pre-test quitter self-identity and quit intention between groups. Extra analyses were conducted to account for these differences.  **Outcome assessment**: 39% of the participants dropped out. Dropouts were significantly younger and had been smoking for fewer years compared to completers.  **Blindness**: Not reported. As the study was conducted online, there was no direct contact between participants and the experimenter. **Adherence**: Manipulation checks and sensitivity analyses were performed. 9% of the participants did not comply with the possible-self exercise.  **Other:** To prevent consistency bias and social desirability bias, different items were used to measure quitter self-identity at post-test compared to pre-test. |
|  | **QUALITATIVE** | | | | |  |
|  | **Qualitative approach** | **Data collection** | **Findings** | **Interpreta-tion of results** | **Coherence of data collection and analysis** |  |
|  |  |  |  |  |  | **Other:** Qualitative data consists of participants’ written responses to the quitter self-identity (possible-self) writing exercise, which is more restricted than in some other qualitative studies. |
|  | **MIXED METHODS** | | | | |  |
|  | **Adequate rationale** | **Integration of components** | **Interpreta- tion of integration** | **Divergences**  **/Inconsis-tencies** | **Overall quality of mixed methods** |  |
|  |  |  |  |  |  | **Adequate Rationale:** Qualitative analysis/mixed-methods approach was not explicitly mentioned, so rationale is unclear.  **Integration of components:** The qualitative results are integrated with the quantitative results in the analyses.  **Interpretation of integration:** In the results and discussion sections, the qualitative and quantitative data are clearly integrated and interpreted together.  **Divergences/inconsistencies:** Not reported.  **Overall quality assessment rating:** 67% |
| **Multi-component interventions** (*n* = 1) | | | | | | |
| Priebe (2020) &  Wierts (2023) | **QUANTITATIVE** (non-randomized) | | | | |  |
|  | **Target population** | **Measure- ments** | **Complete outcome data** | **Confoun-ding variables** | **Intervention as intended** |  |
|  |  |  |  |  |  | **Target population:** Not clearly defined. While it can be inferred from the paper, the in- and exclusion criteria are not explicitly mentioned.  **Measurements:** Suitable objective and self-report measures of parameters were used.  **Complete outcome data:** 50% of the participants dropped out. However, no significant differences were found between dropouts and completers. Intention-to-treat analyses were conducted and compared with observation-only analyses.  **Confounding variables:** Potential predictors of changes in relevant outcome measures are assessed.  **Intervention as intended:** The intervention was consistent with the description in the protocol article, and fidelity was assessed.  **Other:** Author of the secondary analysis paper was contacted about a question regarding the sample and responded with a clarification. |
|  | **QUALITATIVE** | | | | |  |
|  | **Qualitative approach** | **Data collection** | **Findings** | **Interpreta-tion of results** | **Coherence of data collection and analysis** |  |
|  |  |  |  |  |  | **Qualitative approach**: Thematic analysis was used, and the methods appear appropriate.  **Data collection:** Interviews were conducted with both participants (dropouts and completers) and coaches, enriching the data. The questions for the interviews and open-answer questions seem appropriate and complete, although possibly a bit more focused on the positive effects of the intervention (compared to negative).  **Interpretation of results**: The selected quotes fit the theme.  **Coherence:** The number of participants and coaches interviewed, as well as their characteristics, is not reported. Although the methods are described in more detail in another article [2], it is not specified if the qualitative interview sample matches that described in the other paper. |
|  | **MIXED METHODS** | | | | |  |
|  | **Adequate Rationale** | **Integration of components** | **Interpreta-tion of integration** | **Divergences/Inconsis-tencies** | **Overall quality of mixed methods** |  |
|  |  |  |  |  |  | **Adequate rationale**: It is mentioned in the introduction that both quantitative and qualitative methods are used, but this could be more clearly stated in the title and description of the design. The rationale is unclear.  **Integration of components**: It is clear that quantitative and qualitative data were gathered separately and integrated during the results interpretation.  **Interpretation of integration:** In the discussion section, the qualitative and quantitative data are integrated and interpreted together.  **Divergences/Inconsistencies**: Inconsistencies in the qualitative and quantitative findings are explained.  **Overall quality assessment rating:** 74% |

Legend: Green = yes (no indication of bias); orange = can’t tell/no information was given; red = no (indication of bias)

*Notes*.

^a^More information about the biases and criteria can be found in the MMAT manual [1]. Mixed-methods papers were scored for their quantitative and qualitative approaches and the integration of these parts separately.

^b^The final MMAT quality assessment rating ranges from 0% (i.e., all biases rated as ‘no’ or ‘can’t tell’; lowest rating) to 100% (i.e., all biases are rated as ‘yes’; highest rating), with 20% given for each bias scored as ‘yes’. For mixed-methods studies, for which 15 biases were evaluated instead of five, the total number of ‘yes’ ratings was divided by three to calculate the overall rating.

^c^Blinded outcome assessment only refers to whether researchers were blinded to intervention assignment during data collection. None of the articles mentioned whether the analysis was blinded or not.

**Table S4.3.**

MMAT assessment for quantitative non-randomized trials (*n* = 1/20).

| **Article:** First author (year) | **Biases**^a^ |  |  |  |  | **Notes**  Overall quality assessment rating^b^ |
| --- | --- | --- | --- | --- | --- | --- |
| **Interventions challenging identity** (*n* = 1) | | | | | | |
| Strachan (2008) | **Target population** | **Measure- ments** | **Complete outcome data** | **Confoun-ding variables** | **Intervention as intended** |  |
|  |  |  |  |  |  | **Target population**: It is described that the results are generalizable to the population of fitness class participants, not the general population. The focus was on active individuals who already have a strong exercise identity, though in- and exclusion criteria are not explicitly stated.  **Measurements**: Suitable measures of parameters were used and the selection of parameters was based on theoretical foundations.  **Complete outcome data**: Of the interested individuals contacted, 94% completed the questionnaire.  **Confounding variables**: Potential confounding variables were assessed and discussed.  **Intervention as intended**: The intervention was pilot-tested, but no manipulation checks were conducted.  **Overall quality assessment rating:** 80% |

Legend: Green = yes (no indication of bias); orange = can’t tell/no information was given; red = no (indication of bias)

*Notes*.

^a^More information about the biases and criteria can be found in the MMAT manual [1].

^b^The final MMAT quality assessment rating ranges from 0% (i.e., all biases rated as ‘no’ or ‘can’t tell’; lowest rating) to 100% (i.e., all biases are rated as ‘yes’; highest rating), with 20% given for each bias scored as ‘yes’.

**References**

1. Hong QN, Fàbregues S, Bartlett G, Boardman F, Cargo M, Dagenais P, et al. The Mixed Methods Appraisal Tool (MMAT) version 2018 for information professionals and researchers. Educ Inform. 2018;34(4):285-91. <https://doi.org/10.3233/Efi-180221>.

2. Glowacki K, O'Neill M, Priebe CS, Faulkner G. 'When you put the Group and the Running Together ...': A Qualitative Examination of Participant Experiences of the Canadian Run to Quit program. J Smok Cess. 2019;14(1):52-8. <https://doi.org/10.1017/jsc.2018.13>.
